# Long-term exposure to the ethanol-derived metabolite acetaldehyde elevates structural genomic alterations but not base substitutions

**DOI:** 10.1038/s42003-026-09521-1

**Published:** 2026-01-17

**Authors:** Rita Lózsa, Bernadett Szikriszt, Eszter Németh, Zoltán Szeltner, Regina Martinek, Ádám Póti, Timóteus Feik, Sándor Kollarics, Bence G. Márkus, Nnennaya Kanu, Zsófia Sztupinszki, Ferenc Simon, Tatsuhiro Shibata, Charles Swanton, Zoltan Szallasi, Andrea L. Richardson, Dávid Szüts

**Affiliations:** 1 Institute of Molecular Life Sciences, HUN-REN Research Centre for Natural Sciences, Budapest, Hungary; 2 Doctoral School of Biology, ELTE Eötvös Loránd University, Budapest, Hungary; 3 Institute for Solid State Physics and Optics, HUN-REN Wigner Research Centre for Physics, Budapest, Hungary; 4 Department of Physics, Institute of Physics, Budapest University of Technology and Economics, Budapest, Hungary; 5 Stavropoulos Center for Complex Quantum Matter, Department of Physics and Astronomy, University of Notre Dame, Notre Dame, IN USA; 6 Cancer Research UK Lung Cancer Centre of Excellence, University College London Cancer Institute, London, UK; 7 Danish Cancer Institute, Copenhagen, Denmark; 8 Computational Health Informatics Program, Boston Children’s Hospital, Boston, MA USA; 9 Laboratory of Molecular Medicine, The Institute of Medical Science, The University of Tokyo, Tokyo, Japan; 10 Cancer Evolution and Genome Instability Laboratory, The Francis Crick Institute, London, UK; 11 Department of Medical Oncology, University College London Hospitals, London, UK; 12 Department of Bioinformatics, Semmelweis University, Budapest, Hungary; 13 Johns Hopkins University School of Medicine, Baltimore, MD USA; 14 National Laboratory for Drug Research and Development, Budapest, Hungary

**Keywords:** Cancer genomics, Genomic instability, DNA damage and repair

## Abstract

Acetaldehyde is the primary metabolite of ethanol, and routes of exposure include endogenous sources, food and cigarette smoke. To explore whether the mutagenic effect of acetaldehyde is responsible for the carcinogenicity of ethanol, we use whole genome sequencing on four human cell lines subjected to long-term, physiologically relevant, analytically validated acetaldehyde treatments. Unexpectedly, the treatments do not induce increased base substitution and short insertion/deletion mutagenesis, nor the appearance of the alcohol-associated cancer mutation signature SBS16. In contrast, we observe large genomic alterations in most cell lines, which parallel the association of 32 kb to 1 Mb deletions and duplications with alcohol consumption in a Japanese gastric cancer cohort. Observations of DNA damage response and a specific requirement for the homologous recombination pathway to tolerate acetaldehyde suggest that DNA breaks are responsible for structural genomic alterations in both cell line and tumour samples, and these may contribute to the carcinogenic effect of acetaldehyde.

## Abstract (web summary)

Acetaldehyde treatment and whole genome sequencing of human cells reveal no increased base substitution mutagenesis but an induction of structural genomic alterations mirrored by the association of similar events with alcohol consumption in cancer.

## Introduction

Acetaldehyde is classified as a Class I human carcinogen by the International Agency for Research on Cancer^1^. In humans, most acetaldehyde exposure originates from the consumption of alcoholic beverages, but smoking, air pollution and fermented foods are also important sources^2,3^. During ethanol metabolism, the majority of acetaldehyde is formed by alcohol dehydrogenases in the liver^4^. In addition, most alcoholic drinks already contain acetaldehyde with reported average concentrations ranging from 195 μM in beer to 2417 μM in fortified wine^5^. Thus, the highest local concentration during acetaldehyde ingestion occurs in the oral cavity and oesophagus. Accordingly, consumption of alcoholic drinks elevates the risk of oral, laryngeal and oesophageal cancer approximately five-fold in contrast to the two-fold elevation of liver cancer risk, and more modest increases in the risk of stomach, pancreas, colorectal and other cancer types^6^. Notably, alcohol consumption also increases the risk of female breast cancer^7,8^.

Evidence that ethanol exerts its main carcinogenic effect through acetaldehyde comes from the observation that ethanol-related cancer risk is greatly elevated in carriers of the aldehyde dehydrogenase ALDH2*2 (E504K) gene variant, which results in a dramatic loss of activity in this homotetrameric enzyme even when only one subunit is inactive^9^, and causes very high acetaldehyde concentrations even in heterozygous individuals^10^. The genotoxicity of acetaldehyde may explain its carcinogenic effects. Acetaldehyde is capable of inducing DNA adducts, including *N*^2^-ethylidene-2’-deoxyguanosine (ethylidene-G), 1, *N*^2^-propano-2′-deoxyguanosine, as well as DNA-DNA and DNA-protein crosslinks (DPCs)^11–14^.

A history of alcohol consumption was significantly correlated with the appearance of a base substitution mutation signature in several whole-exome sequence datasets of oesophageal cancers^15–17^, which was similar to the SBS16 cancer mutation signature that is dominated by T > C transitions^18^. The contribution of the alcohol-associated signature to each mutation spectrum was minor. SBS16 was also identified in head and neck cancer exome datasets, where it showed a significant association with tobacco but not alcohol consumption^19^. An increased contribution of SBS16 was found amongst *TP53* driver mutations in oesophageal tumours of drinkers (6%) versus non-drinkers (2%), and the same study confirmed the association of SBS16 with drinking in the total genomic mutation set, though it did not have the statistical power to separate the effect of drinking from that of smoking^20^.

In vitro mutagenesis assays with acetaldehyde are made challenging by its low boiling point (20.1 °C at 100 kPa), and the consequent very rapid evaporation from cell culture. Whereas several studies reported biological effects of acetaldehyde at treatment concentrations below 100 μM^21–23^, many others used orders of magnitude higher concentrations in the 10–50 mM range^24–26^ on mammalian cells. Indeed, treatment of human induced pluripotent stem cells with 12 mM acetaldehyde was non-lethal, and whole genome sequencing revealed no extra mutations in the surviving cells^24^. Some of the apparent differences in toxicity are likely explained by the evaporation of acetaldehyde. It is therefore of interest to determine the mutagenicity of acetaldehyde on human cells at controlled, stable and biologically relevant concentrations.

Soon after ethanol ingestion, oral acetaldehyde concentration reaches 100–150 μM and remains at this level for 2–15 min^27–29^. In peripheral blood, acetaldehyde concentrations range from 1.5 μM^30^ to a peak of 20 μM^31^, depending on the quantity of ingested ethanol. The ALDH2 E504K variant—widespread in the Asian population—increases these values to around 60–80 μM even in heterozygous form^10^. In this work, we determined a maximal tolerable acetaldehyde concentration that is in the range of the physiologically relevant values, and used this in long-term treatments of several human cell lines to assess mutagenicity by whole genome sequencing.

## Results

### Long-term acetaldehyde treatments under controlled conditions

**Fig. 1:**
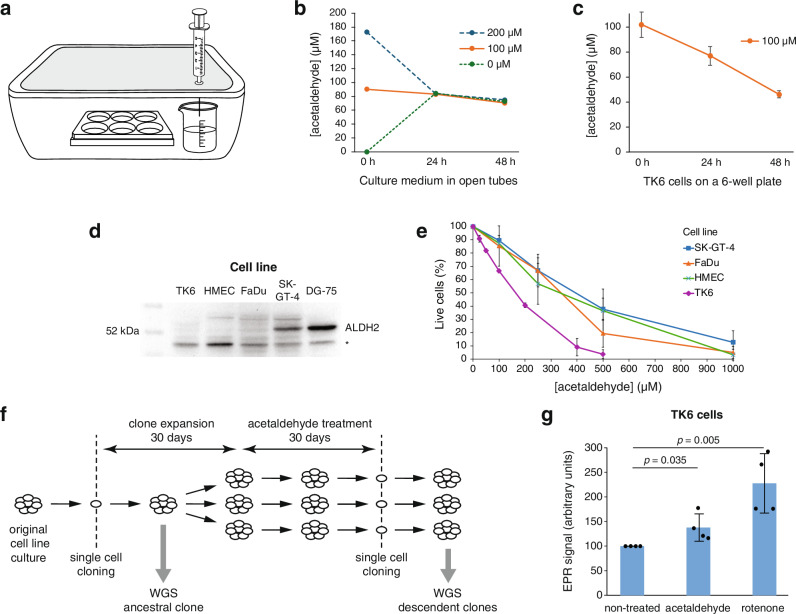
Treatment conditions for long-term acetaldehyde exposure. **a** Enclosed treatment arrangement. Following closure of the box, water with sufficient surplus acetaldehyde is added to equilibrate with the gas phase at a vapour pressure equivalent to 100 μM or any desired concentration at 37 °C. **b** Open tubes of water with the indicated concentration of acetaldehyde were incubated in the treatment box shown in (**a**), and the acetaldehyde concentration was measured at 0 h, 24 h and 48 h. **c** TK6 cells were incubated with an initial concentration of 100 μM acetaldehyde, and the concentration was measured at 0 h, 24 h and 48 h. The mean and S.D. of *n* = 3 independent experiments are shown. **d** Immunoblot of ALDH2 in the four treated cell lines. Human DG-75 cells with strong ALDH2 expression are added as a positive control, whole cell extracts from 100,000 cells are loaded in each lane. A non-specific band is marked with an asterisk. **e** Viability of TK6, HMEC, FaDu and SK-GT-4 cells incubated for 72 h at the indicated starting concentrations of acetaldehyde. Cell viability was measured using an automated cell counter. The mean and S.D. of *n* = 3 independent experiments are shown. **f** Treatment scheme. The oxygen concentration was reduced to 3% between the two cloning steps in the relevant samples. **g** Electron paramagnetic resonance (EPR) measurements of the indicated cell types treated with 100 μM acetaldehyde or 5 μM rotenone for 2 h and incubated with the spin probe CMH for 20 min before collection. Double-integrated EPR signals are normalised to the parallel non-treated sample. Mean, and S.D. are shown, *n* = 4, statistical differences are indicated (ns not significant, unpaired two-sided *t*-test).

We employed an enclosed system for long-term acetaldehyde treatments. Cells were grown on 6-well plates inside a box with acetaldehyde vapour pressure equilibrated to a calculated value from a separate reservoir (Fig. 1a). This set-up ensured minimal losses and equal acetaldehyde concentrations regardless of the starting concentrations (Fig. 1b). Analytical measurements of acetaldehyde levels showed that human TK6 cells experienced gradually decreasing acetaldehyde concentrations (Fig. 1c), apparently slowly metabolising acetaldehyde and accelerating its loss compared to the cell-free control set-up despite the undetectably low expression of ALDH2 (Fig. 1d).

Four human cell lines were chosen to measure the mutagenicity of long-term acetaldehyde exposure. The non-cancer lymphoid TK6 cell line has been frequently used for mutagenicity assays^32^, recently including the use of whole genome sequencing^33,34^. Due to the connection between alcohol and breast cancer, we chose to include the non-transformed HMEC mammary epithelial cells in the study. Complementing the near-euploid TK6 and the euploid HMEC cells, two aneuploid oesophageal cancer cell lines, FaDu and SK-GT-4, were also subjected to treatment (Supplementary Fig. S1).

Treatment with 100 μM acetaldehyde reduced the number of viable TK6 cells by approximately 50% after three days (Fig. 1e). Even though SK-GT-4 cells express ALDH2 at an intermediate level (Fig. 1d), none of the cell lines tolerated 250 μM or 500 μM acetaldehyde over the long term (Supplementary Fig. S2), therefore, we chose a 100 μM concentration for all treatments. An initial clone was isolated from each cell line and expanded for 30 days, followed by 30 days of continuous treatment in a box with or without acetaldehyde with medium changes at each passage (Fig. 1f).

Both ethanol and acetaldehyde have been shown to induce oxidative stress in cultured cells^35,36^, which could contribute to mutagenesis. We used electron paramagnetic resonance (EPR) spectroscopy to directly measure whether 100 μM acetaldehyde treatment generates free radicals; and found a significant increase in the level of radicals and other one-electron oxidants in TK6 cells (Fig. 1g). Lower oxygen exposure reduces the level of reactive oxygen species^37^, therefore long-term acetaldehyde treatments were conducted at both 20% and 3% ambient oxygen to control for this effect.

### Acetaldehyde does not increase base substitutions and short insertions or deletions

**Fig. 2:**
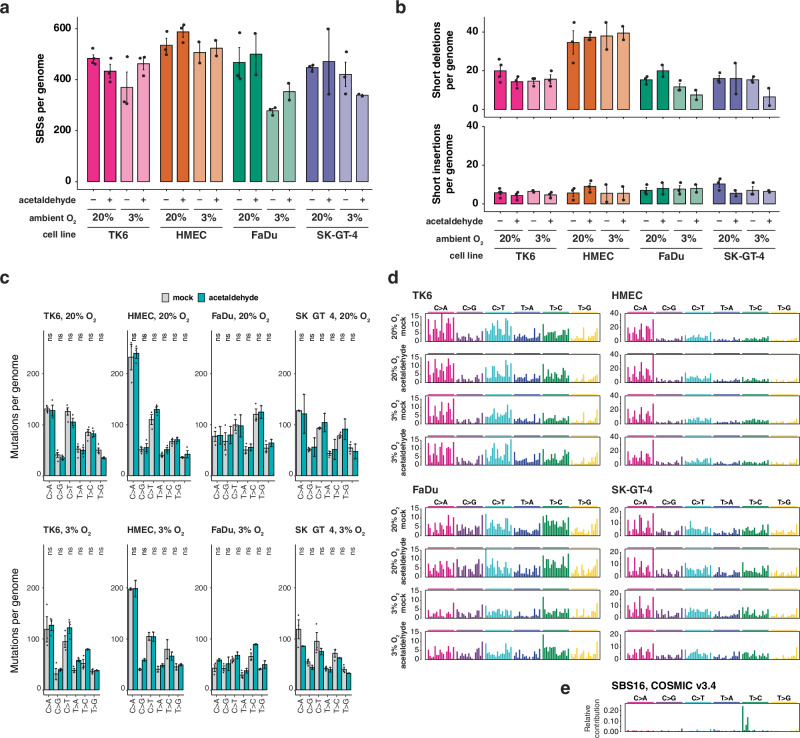
Acetaldehyde does not increase base substitution and short indel mutagenesis. **a** The total number of newly arising single-base substitution (SBS) mutations per sequenced cell line genome, averaged by cell line and treatment condition. Mean and S.E.M. are shown, individual values are indicated. **b** Short deletion and short insertion mutations are shown as in (**a**). **c** Mean and S.E.M. of SBS mutation numbers per genome are shown separately by base change. ns not significant (*p* > 0.05, unpaired two-sided *t*-test with Bonferroni correction for six comparisons). The number of samples (*n*) for all data in (**a**–**c**) is shown in Supplementary Data 3. **d** The 96-category SBS mutation spectra showing the mean number of mutations in each cell type for each treatment type. Each mutation category (e.g. C > A, shown above the panels) is divided into 16 sub-categories based on the identity of the preceding and following base (e.g. ACA > AAA). Base triplets are shown in alphabetical order. **e** The SBS16 mutation signature from version 3.4 of the COSMIC database.

Newly arising mutations were identified in the genomes of single cell clones isolated after the end of the 30-day treatment (Fig. 1f). We detected 400–600 base substitution mutations following the 60-day total culture period in the genomes of each of the four cell lines, and and the inclusion of the acetaldehyde for the last 30 days did not significantly increase the total number of base substitutions (Fig. 2a). The background base substitution rates were slightly lower in 3% O_2_, but acetaldehyde also did not induce extra base substitutions under these conditions (Fig. 2a). Acetaldehyde treatment also did not significantly increase the numbers of newly arising short insertion or short deletion mutations under either 20% or 3% O_2_ in any of the assayed cell lines (Fig. 2b), and dinucleotide mutations were generally also not induced (Supplementary Fig. S3a).

A detailed look at base substitutions by base change did not show significant changes in any of the six categories in any of the cell lines (Fig. 2c; unpaired two-sided *t*-test with Bonferroni correction). As the alcohol-associated SBS16 mutational signature mainly contains T > C mutations, and forms only a minor component of tumour genomes, we also investigated the high-resolution triplet SBS spectra of the sequenced genomes, but we found no clear indication of a treatment-associated increase in the main ATN > ACN SBS16 peaks (Fig. 2d, e). The deconstruction of the spectra to COSMIC SBS signatures also showed no contribution by SBS16 (Supplementary Fig. S3b), which could be found at as low as 5% contribution according to simulations (Supplementary Fig. S3c, d). The deconstructions also did not show an induction of SBS mutations of any other signature, and indicated that none of the tested cell lines possess any clear DNA repair defect^38^. Although SBS mutations were underrepresented in genic regions, we observed no transcriptional strand bias in acetaldehyde-treated samples in any base substitution category, indicating the absence of significantly mutagenic adducts that would be targets of transcription-coupled repair (Supplementary Fig. 4a, b).

### Acetaldehyde elevates the formation of structural variations (SVs)

**Fig. 3:**
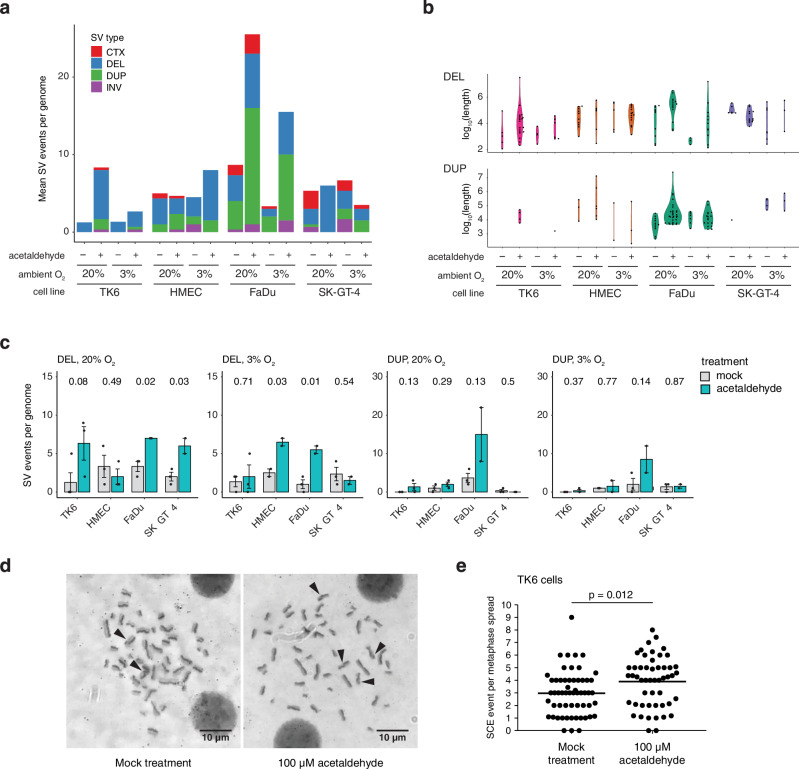
Acetaldehyde elevates the formation of SVs. **a** The mean number of genomic SV events for each cell type and treatment. CTX chromosomal translocation, DEL deletion, DUP duplication, INV inversion. **b** Size distribution of deletions and duplications; each detected event is shown by a black marker. **c** Mean and S.E.M. of deletions and duplications larger than 100 bp. Individual values are shown, the number of samples (*n*) for all data is shown in Supplementary Data 3. Significance values for comparisons between acetaldehyde-treated and control samples are shown above each column pair (unpaired two-sided *t*-test). **d** Representative metaphase spreads of cells labelled for counting sister chromatid exchange (SCE) events. **e** SCE numbers in TK6 cells treated with 100 μM acetaldehyde for 8 h and matched untreated controls. Data from two independent experiments is shown, individual values are normalised to the total number of countable chromosomes, the means are indicated, and the statistical difference is shown (unpaired two-sided *t*-test on the cumulative dataset, *n* = 57 and *n* = 51, respectively).

SVs, which encompass large deletions, large insertions, large duplications, inversions and translocations, were separately identified in all sequenced cell clones. Deletions and duplications were most common amongst these generally rare events (Fig. 3a), with deletions in a broad size range up to 1 Mb identified in all cell lines, and duplications typically between 1 kb and 1 Mb identified primarily in FaDu cells (Fig. 3b). We found a significant increase upon acetaldehyde treatment in >100 bp deletions in the oesophageal FaDu and SK-GT-4 cell lines in 20% O_2_ (Fig. 3c; *p* = 0.02 and *p* = 0.03, respectively, unpaired two-sided *t*-test), and also significant increases in both HMEC mammary cells and FaDu cells in 3% O_2_ (*p* = 0.03 and *p* = 0.01, respectively). There was also a substantial acetaldehyde-induced increase in the formation of deletions in TK6 cells in 20% O_2_ and a substantial increase of duplications in FaDu cells under both oxygen conditions, but this was not significant due to large variation between the samples (Fig. 3c). The deletions and duplications were largely unclustered (Supplementary Fig. S5a). Most acetaldehyde-induced deletions showed short 1–3 bp sequence microhomology between the two breakpoints, suggesting that they were created by classical or alternative non-homologous end joining (NHEJ) mechanisms (Supplementary Fig. S5b). The increase in deletions in all cell lines, including the primary HMEC cells, indicates that this is a general effect of acetaldehyde, not related to any potential genome instability in the employed cell lines.

Sister chromatid exchanges (SCEs) are a form of large recombinational chromosome rearrangement that are not detectable by DNA sequencing as they represent an exchange of identical chromosome segments. We analysed SCEs in TK6 cells using cytological methods (Fig. 3d), and found that 100 μM acetaldehyde treatment for 8 h significantly increased the mean number of SCEs per metaphase from 2.97 in control cells to 3.91 in treated cells (*p* = 0.012, unpaired two-sided *t*-test on the pooled metaphases from two independent experiments, Fig. 3e). These results are in good agreement with those of Ristow and Obe^21^, who observed an induction of SCEs in human lymphocytes upon treatment with as low as 88 μM acetaldehyde. In conclusion, the analysis of the sequence data and SCEs confirms a genotoxic effect of acetaldehyde that is restricted to large rearrangements.

### Homologous recombination (HR) is required for acetaldehyde tolerance

**Fig. 4:**
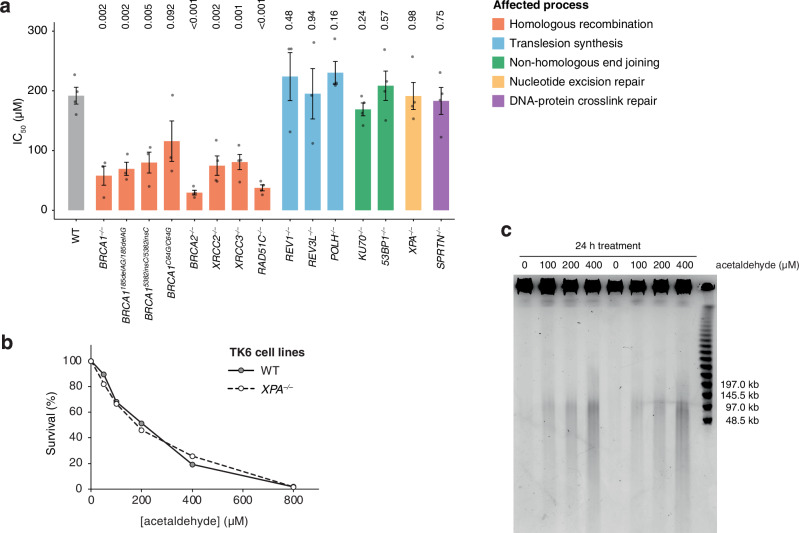
HR is required for acetaldehyde tolerance. **a** Half-maximal inhibitory concentrations (IC_50_) measured after 72-h acetaldehyde treatments in closed tubes in HEPES-buffered growth medium, in a panel of chicken DT40 mutant cell lines with the indicated phenotypes coloured according to the affected DNA repair or damage tolerance pathway. A wide concentration range was used for treatments, and IC_50_ values were determined using normalised dose-response curves fitted with GraphPad Prism. The individual IC_50_ values, means, and S.E.M. are shown, the number of independent repeats (*n* = 3 or *n* = 4) for all data is shown in Supplementary Data 3, and the statistical significance of differences compared to the wild type are indicated (unpaired two-sided *t*-test). **b** Sensitivity of TK6 wild-type and *XPA* knock-out cells to acetaldehyde measured as in (**a**). The mean of *n* = 2 independent experiments is shown. **c** Pulsed field gel electrophoresis of TK6 cells treated with the indicated concentrations of acetaldehyde for 24 h. Two parallel sets of samples are shown.

The lack of SBS and small indel mutagenesis suggests that acetaldehyde-derived DNA lesions are efficiently repaired by DNA repair pathways. We employed an isogenic panel of chicken DT40 cell lines deficient in various DNA repair and bypass pathways to genetically narrow down the type of DNA lesions that may result from acetaldehyde treatment. The half-inhibitory concentration (IC_50_) was determined for each cell line using viability measurements over a concentration series of acetaldehyde in closed tubes in HEPES-buffered culture medium. The IC_50_ was near 200 μM for the wild-type cell line. Knockout mutants of the translesion DNA polymerase genes *REV1*, *REV3L* or *POLH* showed no hypersensitivity (Fig. 4a), suggesting that acetaldehyde treatment does not limit cell growth via replication-stalling lesions. Interestingly, in a recent study, translesion synthesis or base excision repair-deficient TK6 cells showed some sensitivity to short acetaldehyde treatments at high (800 μM) concentrations, which may generate enough adducts to overload the repair pathways^39^. DPC formation has been observed upon acetaldehyde exposure in vitro^40^, and DPCs can be eliminated by transcription-coupled repair or by the SPRTN metalloprotease in a replication-coupled process^41,42^. Nevertheless, neither *XPA* mutants affecting the central factor of both the transcription-coupled and the global genome branch of nucleotide excision repair, nor *SPRTN* mutants showed hypersensitivity to acetaldehyde in DT40 cells, and the knockout of *XPA* also did not have an effect in TK6 cells (Fig. 4a, b).

DNA double-strand breaks (DSBs) can be repaired by the HR and the NHEJ mechanisms. We found that cells mutant for the central HR gene *BRCA2* or for any of the *RAD51* paralogs *XRCC2*, *XRCC3* and *RAD51C* were significantly more sensitive to acetaldehyde than the wild type controls in analogy with an earlier report^43^, *BRCA1* knock-out cells were also hypersensitive, whereas the NHEJ deficient *KU70*^–/^^–^ and *53BP1*^–/–^ cells were not sensitive (Fig. 4a). We also created chicken DT40 mutants affecting the equivalent positions to three common human *BRCA1* inherited founder mutations that increase cancer risk: the 185delAG frameshift mutation affecting the N-terminal RING domain, the C64G missense mutation also in the RING domain, and the 5382insC frameshift mutation in the C-terminal BRCT domain^44^. Each of these homozygous cell lines showed hypersensitivity to acetaldehyde (Fig. 4a), suggesting a strong overlap between the BRCA1 functions required for tumour suppression and acetaldehyde tolerance.

### Acetaldehyde induces DNA breaks and a DNA damage response

**Fig. 5:**
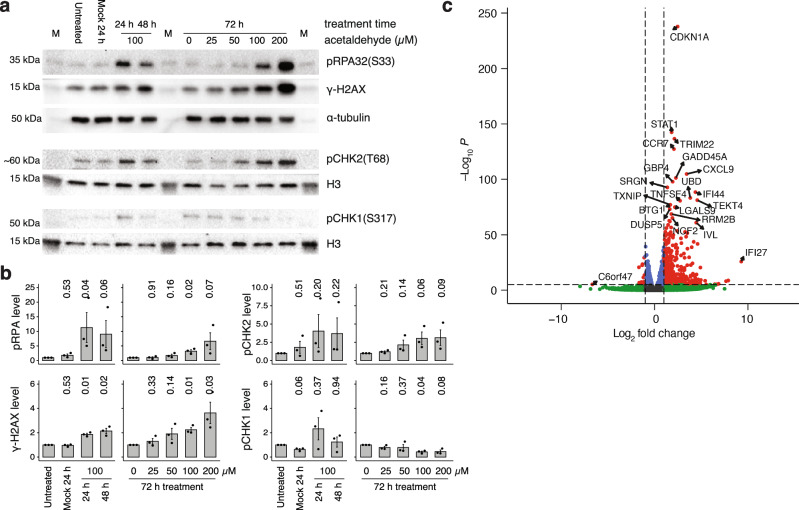
Acetaldehyde induces a DNA damage response. **a** Western blot of whole cell extracts of TK6 cells treated with acetaldehyde as shown, probed against the DNA damage markers γ-H2AX and phospho-RPA32(S33), and the checkpoint activation markers phospho-CHK1(S317) and phospho-CHK2(ST68). An equal number of cells were processed for each lane, loading controls are shown. M size markers. **b** Quantitation of blots from *n* = 3 independent experiments, as shown in (**a**), normalised to the loading control and the untreated sample (marked ‘Untreated’) or zero-dose sample (marked ‘0’). Statistical differences to the normalisation control sample were calculated on the log of relative protein levels using one-sample two-sided *t*-tests; *p* values are shown above the columns. Error bars indicate S.E.M. **c** TK6 cells were treated with 100 μM acetaldehyde for 168 h in triplicate; gene expression changes are shown by fold change and statistical significance of change.

The analysis of DNA repair mutant cell lines suggested that moderate acetaldehyde exposure limits cell proliferation through the formation of DNA breaks rather than DNA adducts. To support this hypothesis, we looked for direct indications of DNA breaks. Neutral pulsed field gel electrophoresis showed a mild but clear and dose-dependent increase in large broken genomic DNA fragments following 100 μΜ acetaldehyde treatment of TK6 cells for 24 h (Fig. 4c). In agreement with this, we observed the dose-dependent appearance of two DNA damage markers, phosphorylated RPA32 (also called RPA2) and phosphorylated histone H2AX. Both markers were significantly elevated upon treatment with 100 μΜ acetaldehyde for 24 or 72 h.(Fig. 5a, b), We also tested the phosphorylation and activation of the checkpoint kinases CHK1 and CHK2, which primarily respond to replication stress and DNA breaks, respectively^45^. We detected a dose-dependent trend in the activation of CHK2, suggesting ATM signalling induced by DNA breaks, but only minor activation of CHK1 at 24 h, which was not sustained after 72 h (Fig. 5a, b). The moderate checkpoint kinase activation is in agreement with a low level of DNA break induction that is compatible with long-term proliferation.

To assess the effect of long-term acetaldehyde treatment on DNA damage response and cell physiology, we performed whole transcriptome sequencing of TK6 cells treated with 100 μΜ acetaldehyde for seven days. The analysis of three independent treated and control samples showed significant induction of the expression of a large number of genes (Fig. 5c), including cell surface proteins and factors of the inflammatory response, but not *ALDH2* (Supplementary Fig. S6a, b). Notably, we observed an approximately 4-fold induction of the *CDKN1A* gene coding for the cell cycle regulator protein p21, and the DNA damage sensor *GADD45A*. Both genes are upregulated in response to DNA damage by p53^46,47^, and p21 induction has indeed been observed in response to acetaldehyde treatment^48^. TK6 is a p53-proficient cell line, and a further p53 target amongst the prominently induced genes was *RRM2B*, a ribonucleotide reductase subunit involved in DNA damage response^49^. The detected transcriptional and post-translational DNA damage responses are in good agreement with the direct observations of DNA breaks, DNA damage checkpoint activation, structural rearrangements and reliance on HR repair.

### Alcohol consumption is associated with genomic SVs in gastric cancers

**Fig. 6:**
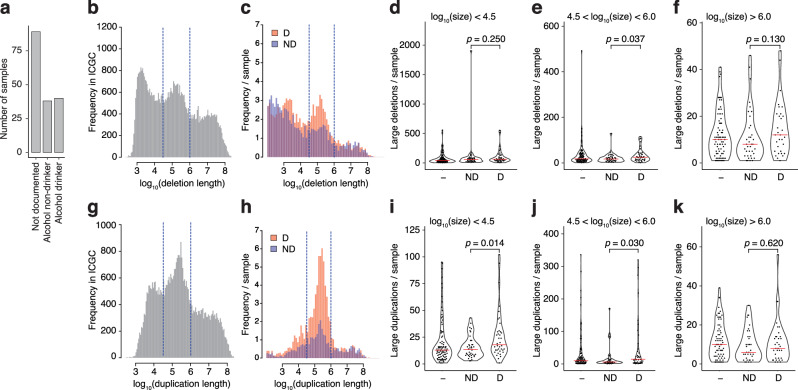
Alcohol consumption is associated with genomic SVs in gastric cancers. **a** Records of alcohol consumption in Japanese gastric cancer patients with analysed genomic SV data. **b** Distribution of large deletion lengths in the available pan-cancer ICGC dataset. **c** Distribution of deletion lengths > 100 bp in Japanese gastric cancer patients classified as drinkers or non-drinkers. **d**–**f** The number of large deletions per gastric cancer genomes according to drinking history, separated by size, is shown above the panels. **g** Distribution of large duplication lengths in the available pan-cancer ICGC dataset. **h** Distribution of duplication lengths > 100 bp in Japanese gastric cancer patients classified as drinkers (D) or non-drinkers (ND). **i**–**k** The number of large duplications per gastric cancer genomes according to drinking history, separated by size, as shown above the panels. The significance of the difference between drinkers and non-drinkers is shown in (**d**–**f**, **i**–**k**) (Wilcoxon rank-sum test).

Finally, we asked whether the acetaldehyde-associated SV mutagenesis is paralleled by a specific increase of genomic SV events in alcohol-associated cancers. A detailed gastric cancer genome dataset has been collected in Japan that includes records of alcohol consumption^50,51^. SVs were mapped in 170 gastric cancer genomes^52^, amongst which 38 cases were classified as non-drinker and 40 cases had a recorded history of alcohol consumption (Fig. 6a). We divided the size range of SVs into three categories based on a trimodal distribution observed in the entire public ICGC dataset in case of both deletions (Fig. 6b) and duplications (Fig. 6g). The gastric cancer dataset showed a distinct peak in samples from drinkers in the middle category of deletions (4.5 < log_10_ *length* < 6, between 32 kb and 1 Mb, Fig. 6c), and the number of deletions was significantly higher in the middle size range in drinkers than non-drinkers (*p* = 0.037, Wilcoxon rank-sum test, Fig. 6d–f). Similarly, duplications in the genomes of drinkers showed a striking peak in the middle size range (Fig. 6h), and the number of duplications was significantly higher in both the smaller and middle size ranges when comparing samples from drinkers and non-drinkers (*p* = 0.014 and *p* = 0.03, respectively, Fig. 6i–k). To test the specificity of this association, we also analysed data from oesophageal adenocarcinomas, which arise at the lower end of the oesophagus and show no association with alcohol consumption^53^. In contrast with gastric cancer, we found no significant alcohol-associated elevation in the number of large deletions or duplications in an oesophageal adenocarcinoma dataset with recorded alcohol consumption information collected by the OCCAMS study in the UK^54^ (Supplementary Fig. S7).

It must be noted that tandem duplications in the above gastric cancer data were also associated with smoking history^52^. Nevertheless, the observed increase in alcohol-associated cancer SV events around the 100 kb size range mirrors the acetaldehyde-induced SV formation observed in our cell line studies, thus, it is possible that ethanol-derived acetaldehyde contributes to their formation during tumorigenesis.

## Discussion

In this work, we assessed the mutagenicity of acetaldehyde in a range of human cell line models. Treatments were performed under enclosed conditions for 30 days, and the concentration of acetaldehyde was tracked by analytical measurements. The analysis of whole genome sequences of post-treatment cell clones clearly showed a lack of base substitution or short indel mutagenesis by long-term acetaldehyde treatment, but an increase above the background rate was seen in the number of SVs in the majority of cell lines.

Mutagenesis may either be the direct consequence of a compound chemically damaging DNA, or an indirect effect through the perturbation of cellular metabolism. Both mechanisms could potentially be relevant in the case of acetaldehyde. The formation of base adducts could be expected to cause base substitution mutagenesis. In the same TK6 cell line as used in the present study, endogenous and exogenous ethylidene-G adducts were separately measured following the addition of isotope-labelled acetaldehyde, and the exogenous adducts only dominated at concentrations of 250 μM and above^55^. Cells therefore regularly experience endogenous aldehyde-derived adducts at similar levels to those caused by exogenous concentrations employed for the long-term tolerable treatments in this study. According to these observations, exogenous acetaldehyde should not be expected to cause specific novel mutational processes, but it may instead elevate components of generic background mutagenesis. Such an effect was indeed observed, but restricted to SV events, including large deletions and large duplications that were similar in size but more numerous in treated than in untreated cells. Significant increases were seen in three of the four tested cell lines, which parallels the significantly higher number of large sub-megabase duplications and deletions seen in gastric cancers of drinkers versus non-drinkers.

We did not find any trace of the base substitution signature SBS16 in the genomes of acetaldehyde-treated cells. What could then cause SBS16 in alcohol-associated cancers? SBS16 is correlated with both alcohol consumption and tobacco smoking, though these correlations are not fully clear and separable^19,20^, and SBS16 showed a specific correlation with the combination of alcohol consumption and smoking in a large head and neck cancer dataset^56^. It is likely that SBS16 is related to aldehyde damage, as it shows particularly high contribution to the mutation burden of gastric cancers in patients with ALDH2 mutations plus a history of alcohol consumption^51^. However, the responsible mutagen may be an aldehyde different from acetaldehyde. Cigarette smoke contains a mixture of different aldehydes^57^, and ethanol could also trigger lipid peroxidation through the formation of reactive oxygen species^58^, leading to the formation of various aldehydes, including crotonaldehyde, which can also be substrates of ALDH2 and potentially damage DNA. The formation of reactive oxygen species was modest in the cell line experiments, which may not be a perfect model for the indirect effects of acetaldehyde due to the closely controlled culture environment. In any case, alcohol-associated cancers do not typically show a hypermutator phenotype, and SBS16 is only present as a minor component of somatic mutations^20^. Therefore, even if SBS16 results from an alcohol-related process, it is very unlikely to significantly contribute to tumorigenesis.

What could be the mechanism of acetaldehyde-induced genomic instability? The observed double-stranded DNA breaks and DNA damage response, together with the analysis of the deletion sequence context, imply that the deletions are formed by NHEJ at DNA DSBs. However, the specific sensitivity of HR mutants but not NHEJ mutants to acetaldehyde suggests that the breaks are primarily substrates for HR that efficiently repairs them in HR-proficient cells in an error-free manner, and NHEJ only handles a very small number of breaks resulting in less than one deletion per cell cycle under tolerable acetaldehyde treatment. Selective reliance on HR-dependent DSB repair is typical in the case of treatments that result in the formation of single-ended DSBs at collapsed replication forks, such as inhibitors of topoisomerase I or PARP-1^59^. Indeed, the pattern of relative acetaldehyde sensitivities seen in our study is very similar to that observed with PARP inhibitors on a similar DT40 cell line panel^60^, and fork collapse has been detected using DNA fibre assays in BRCA2-deficient human cells upon acetaldehyde treatment^61^. This hypothesis is in good agreement with the mutational outcome, as collapsed forks can lead to large deletions or tandem duplications^62,63^. The formation of such events could also rely on break-induced replication and alternative end joining, but we did not genetically test the involvement of these pathways. The cause of fork collapse could either be indirectly generated replication stress and fork stalling, also reported in the case of ethanol treatment of cultured human cells^64^, or collision with replication-stalling DNA lesions. We observed signs of replication stress in the form of dose-dependent RPA phosphorylation, but the lack of sustained CHK1 activation may suggest a downregulation of the replication checkpoint that allows long-term proliferation under the treatment conditions. Candidate replication-stalling lesions include interstrand crosslinks repaired by the Fanconi pathway, as chicken DT40 cells deficient in Fanconi genes are hypersensitive to acetaldehyde^65^, and mouse cells mutant for *Fancd2* show an increased number of mutations and genomic rearrangements due to endogenous aldehydes when *Aldh2* is disabled^43^.

If the carcinogenic effect of ethanol consumption is linked to genotoxicity, it is therefore likely to involve the formation of structural rearrangements as observed both in cultured cells and in a gastric cancer cohort. It is possible that some of the carcinogenic effects of ethanol are indirect and do not increase the mutation burden^66^, instead promoting the growth of cells with pre-existing tumorigenic mutations in analogy with the effect of air pollutants on lung adenocarcinoma^67^. The availability and analysis of further cancer genome datasets with detailed information on both alcohol consumption and confounding factors can bring clarity to the mechanism of ethanol-induced tumorigenesis.

The presented genomic analyses clearly show that chronic exposure of cultured human cells to acetaldehyde elevates the formation of large duplications and deletions without any significant impact on base substitution and short indel mutagenesis. The study was performed on cell types relevant to alcohol-induced carcinogenesis, and comprised controlled long-term treatments under analytically validated conditions that best approximate physiological exposure to acetaldehyde, a toxic compound derived from exogenous sources such as metabolised ethanol, smoke or foodstuffs, and also from normal metabolism. In a gastric cancer cohort, we found structural genomic alterations similar to those detected in the acetaldehyde-treated cell line genomes, and the number of such deletions and tandem duplications was significantly higher in patients with a history of alcohol consumption. Acetaldehyde tolerance requires the HR machinery, and our results suggest that the carcinogenic effect of ethanol and acetaldehyde is at least partially explained by exposure-induced genomic rearrangements.

## Methods

### Cell lines and cell culture

TK6 (JCRB, Ibaraki, Japan, #JCRB1435), FaDu (LGC Standards, Teddington, Middlesex, UK, #ATCC-HTB-43) and SK-GT-4 (Merck, Darmstadt, Germany, #11012007) were cultured in RPMI-1640 medium (Thermo Scientific, Gibco, Waltham, MA, USA, #21875-034) supplemented with 10% foetal bovine serum (Life Technologies Hungary Ltd., Budapest, Hungary, #10270106), 1% penicillin-streptomycin (Lonza, Basel, Switzerland, #17-602E). hTERT-HMEC cells (Clonetech, Mountain View, CA, US) were grown in MEGM Mammary Epithelial Cell Growth Medium BulletKit (Lonza, Basel, Switzerland, #CC-3150). DT40 cell lines were kept in RPMI-1640 medium with 7% foetal bovine serum, 3% chicken serum (Merck, Darmstadt, Germany, #C5405) and 1% penicillin-streptomycin. All cell cultures were maintained in a humidified incubator at 37 °C with 5% CO_2_. Cell lines were tested for mycoplasma contamination and authenticated using the whole genome sequence data.

### Generation of mutant cell lines

BRCA1^185delAG/185delAG^, BRCA1^C64G/C64G^, and BRCA1^5382insC/5382insC^ homozygous point mutant cell lines were generated in the chicken DT40 cell line using CRISPR-Cas9 gene targeting combined with homologous templates. Target sites and sequencing primers are listed in Supplementary Data 1. Oligonucleotides encoding guide RNAs were cloned into the pSpCas9(BB)-2A-GFP vector (Addgene #48138, Watertown, MA, USA). DT40 cells were transfected with 500 ng of the corresponding CRISPR-construct using 4D-Nucleofector with SF Cell Line transfection reagent (Lonza Group AG, Basel, Switzerland) with program CN-150. In case of site-specific mutagenesis of BRCA1^185delAG^ and BRCA1^5382insC^, 1 μl of 100 μM ssDNA template (Integrated DNA Technologies, Coralville, IA, USA) was added to the transfection. The sequences of the ssDNA templates are listed in Supplementary Data 1. The 24 h following transfection, GFP+ cells were selected, and single cells were seeded using limiting dilution. In case of site-specific mutagenesis of BRCA1^C64G^, 500 ng pBlueScript-BRCA1_C64G_pLoxPuro homology directed repair (HDR) plasmid and 500 ng sgRNA-containing pSpCas9(BB)-2A-GFP plasmid were added to the transfection. The HDR plasmid contains 650 bp-long homology arms of the chicken BRCA1 gene modified with the relevant C64G (TGT–GGT) mutation in exon 5. The primers required for HDR plasmid assembly are listed in Supplementary Data 1. Removable *pLox*-Puromycin selection cassette^68^ was inserted into the plasmid using a *BamHI* restriction site that was inserted into intron 5 of chicken BRCA1 using site-directed mutagenesis. Clones were grown for approximately 14 days, in 0.5 μg/ml puromycin (Gibco #A11138-02) where applicable, followed by genomic DNA isolation and amplification of the corresponding genomic CRISPR-target site using Phire Tissue Direct PCR Master Mix (Thermo Fisher Scientific, Waltham, MA, USA). Clones affected by mutagenesis were identified using allele-specific qPCR (TB Green Premix Ex Taq II, TaKaRa, San Jose, CA, USA) using qPCR primers that are listed in Supplementary Data 1. Desired successful site-directed mutagenesis of genomic *BRCA1* was confirmed by Sanger sequencing (Microsynth GmbH, Vienna, Austria). Chromatograms were analysed with the Indigo tool (GEAR-Genomics, https://www.gear-genomics.com). The puromycin selection cassette was removed using transient expression of Cre recombinase.

### Residual acetaldehyde measurement

We used the method of Guan et al. ^69^ with minor adaptations. Ice-cooled and thoroughly mixed cultures were centrifuged at 1500 × *g* for 5 min at 4 °C to remove cells, and 100 μl was withdrawn into ice-cooled tubes. Samples were first deproteinised with 0.5 volumes of 11.7 M (70%) perchloric acid. The pH of the solution was adjusted immediately to 4.0 with 2.4 volumes of 4 M sodium acetate buffer pH 9.0, followed by centrifugation at 2000 × *g* for 10 min at 4 °C, and the supernatant was transferred into a pre-chilled tube. The 2-4-dinitrophenyhydrazine (DNPH) (Merck, Darmstadt, Germany, #D199303) was freshly dissolved in 6 N HCl to a 15 mM (3 mg/ml) final concentration. Acetaldehyde in the sample was derivatised with about >80-fold molar excess of DNPH for 1 h at 22 °C while shaking on a rotary device. The reaction was stopped by adding 3 volumes of 5 M sodium acetate buffer, pH 9.0. Two volumes of acetonitrile were added to the reaction mixtures, and the tubes were vigorously shaken for 1 h at 4 °C while acetaldehyde-DNPH and excess DNPH were extracted into the organic phase. The organic phase was separated by centrifugation at 10,000 × *g* for 5 min at 4 °C, and the solvent was evaporated either under vacuum or under a chemical hood overnight. The dried material was dissolved in 300 μl final volume, first adding pure acetonitrile and gradually supplementing the mixture with water up to a 40:60 acetonitrile/water ratio. The 50 μl of the dissolved material was analysed on an ÄKTA-HPLC system equipped with a P-900 pump, a UV-900 detector and a Jupiter Proteo (Phenomenex, Torrance, CA, USA) C12 column (25 cm × 4.6 mm, 4 μm). The flow rate was 0.7 ml/min, and detection of acetaldehyde-DNPH and DNPH was performed at 360 nm. The 220 nm peptide bond was also monitored to confirm sample deproteinisation. The mobile phase was as follows: A, 0.1% TFA/water (*v*/*v*), and B, 0.1% TFA/acetonitrile (*v*/*v*). The column was equilibrated with 40% B/60% A for 1 column volume (CV), followed by sample injection and a 1 CV elution of unbound materials. The bound material was eluted using a fast linear gradient of 40–100% B within 2.8 CV, followed and re-equilibration at 40% B in 1.5 CV. The retention times (tR) of AA-DNPH and DNPH were confirmed by comparison with standard runs of the sole reagent and the acetaldehyde + reagent materials of increasing acetaldehyde. The values of the areas for the AA-DNPH product peak (tR = 12.37 ml) and for the excess reagent peak (tR = 8.3 ml) under the curve were determined using the integration module of the UNICORN software (GE Healthcare). The peak of the excess reagent was used as an internal standard in correcting the slight differences in recoveries from the dried materials. A calibration curve was constructed by incubating different acetaldehyde concentrations (up to 100 μM) in RPMI-1640 in tightly-closed flasks for 2 h at 37 °C, then withdrawing 100 μl and processing as described above. The area of the 12.37 ml retention peak increased with increasing acetaldehyde concentration. The corresponding area of the absorption curve in the acetaldehyde-free medium control was subtracted. The standard curve of acetaldehyde detection was almost perfectly linear up to 100 μM, as reported previously in ref. ^69^.

### Acetaldehyde cytotoxicity assay

Assays were performed in the appropriate medium buffered with 20 mM K-HEPES pH 7.4 (Merck, Darmstadt, Germany, #54457). As acetaldehyde is highly volatile, suspension cells were treated in 1.5 ml screw cap tubes for 3 days on a horizontal shaker at 50 rpm with initial cell counts of 5000/ml for DT40 and 3000/ml for TK6 at 37 °C. The adherent cells were treated in 12.5 cm^2^ tissue culture flasks with closed plug seal caps for 3 days at 37 °C with initial cell counts of 10,000/ml. On the third day, three technical replicates were pipetted from each tube onto 96-well plates, and viable cell number was measured by using PrestoBlue reagent (Thermo Scientific, Waltham, MA, USA, #A13262). Alternatively, live cells were counted after trypan blue staining using a ViCell automated cell counter (Becton Dickinson, Franklin Lakes, NJ, USA).

### Long-term acetaldehyde treatments

Cells were cultured for 30 days at 20% or 3% O_2_ after a cloning step, then cells from a single clone were seeded into 6-well plates in three independent replicates. The initial cell count was 30,000/ml for TK6 and 30000/well for adherent cells. Cultures were passaged every three and four days for 30 days, adjusted to the initial cell counts; acetaldehyde was supplemented freshly at each passage. All cultures were treated with 100 μM acetaldehyde in an air-tight box, which was previously equilibrated to the atmosphere of the cell culture incubator at 5% CO_2_ and 20% or 3% O_2_. The acetaldehyde concentration was kept constant by calculating the vapour pressure during the treatments, utilising the equilibrium between the cell culture media and an acetaldehyde-containing reservoir. After the 30 days of treatment, single-cell clones were isolated from all replicate cultures, and DNA was extracted when cultures had grown to a sufficient cell number.

### EPR spectroscopy measurements

Two million treated (5 h 100 μM acetaldehyde or 5 μM rotenone) and control TK6 cells were collected and washed with PBS-DTPA (137 mM NaCl, 270 μM KCl, 10 mM Na_2_HPO_4_, 180 μM KH_2_PO_4_ and 1 mM DTPA), then incubated in 2 mM CMH-HCl (Enzo Life Sciences Inc., Farmingdale, NY, USA, #ALX-430-117-M010) in PBS-DTPA for 20 min 200 μl cell suspension was loaded into the bottom of a 5 mm diameter defect-free quartz ESR tube (Wilmad-LabGlass, Vineland, NJ, USA, #710-SQ-250M) with a long needle, snap frozen in liquid nitrogen and stored at −80 °C until measurement. EPR measurements were carried out on a Bruker Elexsys E500 X-band (0.34 T, ~9.4 GHz) spectrometer equipped with the standard Bruker SHQE cavity (Bruker, Karlsruhe, Germany) and evaluated as described earlier in ref. ^37^.

### Whole genome sequencing and mutation detection

DNA was extracted from 1 million cells using the Puregene Cell Kit (Qiagen, Venlo, The Netherlands, #158043). Library preparation and whole genome sequencing were done at Novogene (Beijing, China) using 2× 150 bp paired-end format sequencing on the Illumina platform with 30x mean coverage. Sequencing reads were aligned to the reference genome GRCh38/hg38 using the Burrows-Wheeler alignment algorithm^70^, followed by post-processing with the IndelRealigner tool of the Genome Analysis Toolkit (GATK, version 3.8)^71^.

Single base substitution (SBS) and short indel (<50 bp) mutations were identified using the IsoMut method developed for multiple isogenic samples^72–74^. In brief, after applying a base quality filter of 30, data from all samples in the batch were compared at each genomic position and filtered using optimised parameters of minimum mutated allele frequency (0.2), minimum coverage of the mutated sample (5) and minimum reference allele frequency of all the other samples (0.93). Cell lines with a stable diploid genome (TK6 and HMEC) were analysed in a single IsoMut run, and the mutations in cell lines with large aneuploid regions (FaDu and SK-GT-4) were called in a separate IsoMut run. The final input set for IsoMut runs is listed in Supplementary Data 2. Hits were filtered using a probability-based quality score calculated from the mutated sample and one other sample with the lowest reference allele frequency^72^. During the postprocessing of raw mutation calling data, a minimum threshold was set for the score determined by IsoMut, which was 2.5 in case of SBS mutations, 2.0 for insertions and 2.0 for deletions. Mutation numbers and SBS triplet spectra were determined for each sample and plotted using standard tools in R.

SVs were detected using GRIDSS v2.8.3^75^, run separately for all cell lines and all conditions. In a single GRIDSS run, the genome of 2–4 subclones was compared to the relevant starting clone (see Fig. 1e). The output was subjected to post-filtering using a respective panel of normals. Events were then classified into five subgroups, including chromosome translocations, insertions, deletions, inversions and duplications. Hits with low support and hits that are not unique in the GRIDSS run were removed. As a final step, all events were checked in the IGV browser. Since IsoMut, optimised for small indels and SBSs, and GRIDSS, specialised for large genomic events, do have an overlap in size range, the results were merged. The events found by both software were included in the analysis of small events, and SVs found only by GRIDSS were subjected to separate analysis.

Genome-scale sequence coverage was plotted by determining the coverage at every 10-kbp position in the genome using *samtools bedcov*. A moving average using 100 neighbouring data points was shown for clarity. In parallel, the B allele frequency of an SNP list of the human reference genome containing 241,034 positions was also determined for the starting clone.

Transcription strand bias was determined based on SNV annotation with *TxDb.Hsapiens.UCSC.hg38.knownGene* using the *mut_strand* function of *MutationalPatterns*^76^.

### Mutational spectrum deconstruction

Mutational spectrum deconstruction was performed with the *fit_to_signatures_strict* function of *MutationalPatterns*^76^, with the COSMIC signature reference set 3.1. Maximal delta value was increased to 0.08 to avoid overfitting using more than 10 signatures, providing cosine similarity greater than 0.93 for all samples, which is acceptable for samples with low mutation numbers. Sensitivity of detection was assessed by the sequential addition of an increasing number of random mutations with the COSMIC SBS16 spectrum to the total set of mutations observed in mock-treated TK6 samples. The same maximal delta value assured cosine similarities greater than 0.96.

### Western blot

Cells were boiled for 5 min in 1 volume of 4× Laemmli buffer (200 mM Tris, pH 6.8, 400 mM DTT, 8% SDS, 40% glycerol, 0.08% (*w*/*v*) bromophenol blue) and resolved in a 10% SDS-PAGE. Following semi-dry transfer, 0.2 μM pore size PVDF membranes (Bio-Rad, Hercules, CA, USA, #1620177) were blocked in 5% non-fat milk or in 5% BSA in TBST (20 mM TRIS pH 7.6 and 150 mM NaCl, 0.1% Tween 20). Anti-ALDH2 antibody (Proteintech Europe, Manchester, UK, #15310-1-AP), phospho-RPA32 (Ser33) antibody (Novus Biologicals, Bio-Techne, Minneapolis USA, #NB100-544), phospho-histone H2A.X (Ser139) antibody (Sigma-Aldrich, Missouri, USA, #ZMS05636), phospho-Chk1 (Ser317) antibody (Cell Signalling Technology, Leiden, The Netherlands, #2344S) and phospho-Chk2 (Thr68) antibody (Cell Signalling Technology, Leiden, The Netherlands, #2661), H3-histone antibody (Santa Cruz Biotechnology, Dallas, USA, #sc-10809) and α-tubulin antibody (Merck, Darmstadt, Germany, #T6199) were applied at 1:1000 dilution for 1 h in 2.5% non-fat milk or in 2.5% BSA in TBST and after washing, membranes were probed with an anti-rabbit IgG-HRP secondary antibody (Merck, Darmstadt, Germany, #A0545) or anti-mouse IgG-HRP secondary antibody (Merck, Darmstadt, Germany, #A9044) at 1:10,000 dilution. Clarity Western ECL Substrate (Bio-Rad, Hercules, CA, USA, #102031863) was applied to the membranes, and chemiluminescent signal was detected by a ChemiDoc MP Imager (Bio-Rad) and analysed using the ImageLab software (Bio-Rad).

### SCE assay

Two and five-tenths million TK6 cells were incubated for 28 h in 30 ml RPMI-1640 growth medium containing 20 μM BrdU (Merck, Darmstadt, Germany, #B5002). Hundred micromolar acetaldehyde was added for 8 h, and cells were arrested in metaphase by adding 0.1 μg/ml colcemid for 3 h before harvesting the samples. After adding the acetaldehyde, the culture flasks were sealed. Cells were collected by centrifugation for 5 min at 250 × *g* and hypotonised for 20 min with 75 mM KCl at room temperature. Swollen cells were centrifuged at 250 × *g* for 5 min and slowly resuspended with ice-cold methanol:glacial acetic acid (3:1), then repeatedly centrifuged at 250 × *g* for 5 min at 4 °C and resuspended in smaller volumes (20 ml, 15 ml, 5 ml, and 2 ml). Samples were stored at 4 °C.

Clean, humid and pre-chilled slides were placed at a 30–45° angle, and 30 μl cell droplets were dropped onto them from 20 to 30 cm. After 20 min of incubation at 50 °C on a dry bath block, the slides were stained with 5 μg/ml Hoechst 33258 (Merck, Darmstadt, Germany, #B1155) diluted in 2 × SSC (pH 7.0) for 20 min at room temperature. The slides were washed in McIlvaine buffer (pH 8.0), and coverslips were placed on them to prevent drying out. Slides were exposed to 365 nm UV with one 8-Watt UVP lamp from ~7 cm for 30 min while incubating at 50 °C. Following a 1-h incubation in 2× SSC at 50 °C, slides were rinsed by immersing in distilled water and stained with the Shandon Kwik-Diff stain kit (Thermo Scientific). After staining, the slides were rinsed with lightly running tap water and left to dry. Coverslips were mounted with DPX (Merck, Darmstadt, Germany, #06522). Counting was performed on a Leica DM IL LED Inverted Fluorescence Microscope using a 100× oil-immersion objective.

### Pulsed field gel electrophoresis

Cells were harvested after the required acetaldehyde treatment, pelleted at 1000 g, resuspended in TE, and then mixed with molten 1.2% low-melting-point agarose (AgarPlaque Plus^TM^ Agarose, Cat. #: 554766) 1:1. The suspension was carefully pipetted into a PFGE plug mould, 60 µl per plug, and cooled to 4 °C. The solid plugs were submerged in 100 µl digestion solution (0.5 M EDTA, 1 *w*/*v*% *N*-laurylsarcosine, 1 mg/ml Proteinase K, 10 mg/ml RNase A) in a 1.5 ml centrifuge tube, then placed in a 50 °C dry bath. After 24 h, the inserts were loaded into wells of a 1% agarose gel (SeaKem LE Agarose, Cat.#: 50004) cast with the Bio-Rad CHEF-DRII gel casting platform. The samples were run in the Bio-Rad CHEF-DRII PFGE system, with 4 V/cm, 14 °C, initial/final switch time 5 s/120 s, in 0.5× TAE buffer for 24 h. After the run, the gel was stained with ethidium bromide for 30 min prior to imaging with a Bio-Rad ChemiDoc^TM^ MP Imaging system.

### RNA isolation and processing of RNA-seq data

One million TK6 cells were harvested after 7-day treatments (mock or 100 μM acetaldehyde). Cells were pelleted at 1000 rpm, washed twice with PBS and resuspended in 1 ml ice-cold TRI Reagent (Sigma-Aldrich, Missouri, USA, #T9424). After adding 180 μl ice-cold chloroform, the samples were mixed for 20 s on a vortex shaker followed by 5 min on ice. The samples were fractionated by centrifugation at 14,000 rpm for 20 min at 4 °C. RNA was precipitated from the supernatant with 400 μl ice-cold isopropanol, followed by mixing and incubation on ice for 15 min. RNA pellets were collected by centrifuging at 14,000 rpm for 20 min at 4 °C. RNAs were washed with 800 μl 80% ice-cold ethanol, followed by centrifuging at 11,500 rpm for 10 min at 4 °C. The isolated RNA pellets were dried for 10 min, then dissolved in 30 μl sterile nuclease-free water via incubation at 60 °C for 10 min. The genomic DNA was eliminated from the total RNA samples with 2.5 M lithium chloride precipitation (Thermo Fisher Scientific, Waltham, MA, USA, #AM9480). The quality control of the RNAs was assessed by agarose gel electrophoresis and Nanodrop-2000 measurements (Thermo Fisher Scientific, Waltham, MA, USA). RNA samples were sequenced by Novogene (UK) on the NovaSeq X Plus platform in 2 × 150 bp paired-end format, providing 6 Gb of raw data per sample.

Abundances of transcripts were calculated using *kallisto 0.48.0*^77^ from fastq files, and were imported and summarised with *tximport*^78^. Genes were annotated based on the Ensembl genes build GRCh38.111. Differentially expressed genes were determined with *DESeq2*^79^. Approximate Posterior Estimation for generalised linear model (R package *Apeglm*^80^) was used for log fold change shrinkage to reduce the noise of low-expression genes. A volcano plot was generated using the R package *EnhancedVolcano*. The *p* value cutoff was 10^−5^, and the log_2_ fold change cutoff was 1. For checking *ALDH2* expression, raw sequencing reads were also aligned with *HISAT2*^81^, version 2.1.0. GRCh38 was used as a reference genome. Alignments were displayed in *Integrative Genomics Viewer*. Pathway analysis was done with the *gseGO* utility of *clusterProfiler v3.0. 4*^82^, using ENSEMBL keytype, nPerm = 10000, minGSSize = 3, maxGSSize = 800, pvalueCutoff = 0.05. The *Dotplot* function of *enrichplot* was used to visualise the results.

### Cancer data analysis

SVs detected in gastric cancer whole genome sequence data^52^ were analysed based on the alcohol drinking history. For the analysis, SV events, including duplications and deletions, were categorised into three groups, based solely on the length of the event. The three categories were log_10_(SV length) < 4.5, 4.5 < log_10_(SV length) < 6.0, and log_10_(SV length) > 6.0. These categories were set based on the trimodal distribution of SVs across all samples with uncontrolled access in the ICGC data set^83^. For this, data were downloaded from the ICGC Portal, and samples from all tissues were analysed together for SV length, comprising 179,968 SV events in 1816 samples from 1727 donors in 25 sequencing projects. Due to the low number of samples with adequate metadata information regarding alcohol consumption, only the ESAD-UK data set^54^ was analysed in detail. The non-parametric Wilcoxon rank-sum test was applied to compare the number of events between alcohol drinking and non-drinking patients.

### Statistics and reproducibility

Sample sizes for genomic analyses were almost universally *n* = 3 independent biological cell clones cultured at the same time. Replicates in all other experiments were derived from independent experiments performed at different times, the number of replicates is reported throughout. The standard deviations and the expected effects were not known in advance, therefore, *n* = 3 or *n* = 4 was chosen for all experiments. Unpaired two-sided *t*-tests were typically used for the comparison of experimental data, with Bonferroni correction where multiple comparisons on the same dataset were performed with an exploratory approach (Fig. 2c). One-sample *t*-test was used for data normalised to the control (Fig. 5b). Nonparametric tests were used on patient-derived genomic data.

### Ethics statement

This manuscript reports the analysis of previously published human cancer data with appropriate ethics approval^51,52^.

### Reporting summary

Further information on research design is available in the Nature Portfolio Reporting Summary linked to this article.

## Supplementary information

Supplemental Information

Description of Additional Supplementary Files

Supplementary Data 1

Supplementary Data 2

Supplementary Data 3

Reporting summary

## Data Availability

Source data for all figures is provided in Supplementary Data 3. Uncropped blots are shown in Supplementary Fig. S8. Whole genome sequence data obtained for this study are available from the European Nucleotide Archive under study accession number PRJEB81715. All other data are available from the corresponding authors on reasonable request.

## References

[1] Secretan, B. et al. A review of human carcinogens–part E: tobacco, areca nut, alcohol, coal smoke, and salted fish. *Lancet Oncol.* **10**, 1033–1034 (2009). 19891056 10.1016/s1470-2045(09)70326-2

[2] Salthammer, T. Acetaldehyde in the indoor environment. *Environ. Sci. Atmos.* **3**, 474–493 (2023).

[3] McLaughlin, S. D., Scott, B. K. & Peterson, C. M. The effect of cigarette smoking on breath and whole blood-associated acetaldehyde. *Alcohol* **7**, 285–287 (1990). 2390202 10.1016/0741-8329(90)90083-o

[4] Cederbaum, A. I. Alcohol metabolism. *Clin. Liver Dis.* **16**, 667–685 (2012). 23101976 10.1016/j.cld.2012.08.002 PMC3484320

[5] Lachenmeier, D. W. & Sohnius, E. M. The role of acetaldehyde outside ethanol metabolism in the carcinogenicity of alcoholic beverages: evidence from a large chemical survey. *Food Chem. Toxicol.* **46**, 2903–2911 (2008). 18577414 10.1016/j.fct.2008.05.034

[6] Bagnardi, V. et al. Alcohol consumption and site-specific cancer risk: a comprehensive dose-response meta-analysis. *Br. J. Cancer* **112**, 580–593 (2015). 25422909 10.1038/bjc.2014.579 PMC4453639

[7] Sun, Q. et al. Alcohol consumption by beverage type and risk of breast cancer: a dose-response meta-analysis of prospective cohort studies. *Alcohol Alcohol* **55**, 246–253 (2020). 32090238 10.1093/alcalc/agaa012

[8] Zhang, S. M. et al. Alcohol consumption and breast cancer risk in the Women’s Health Study. *Am. J. Epidemiol.* **165**, 667–676 (2007). 17204515 10.1093/aje/kwk054

[9] Weiner, H., Wei, B. & Zhou, J. Subunit communication in tetrameric class 2 human liver aldehyde dehydrogenase as the basis for half-of-the-site reactivity and the dominance of the oriental subunit in a heterotetramer. *Chem. Biol. Interact.* **130-132**, 47–56 (2001). 11306030 10.1016/s0009-2797(00)00221-0

[10] Peng, G. S., Chen, Y. C., Wang, M. F., Lai, C. L. & Yin, S. J. ALDH2*2 but not ADH1B*2 is a causative variant gene allele for Asian alcohol flushing after a low-dose challenge: correlation of the pharmacokinetic and pharmacodynamic findings. *Pharmacogenet. Genomics* **24**, 607–617 (2014). 25365528 10.1097/FPC.0000000000000096

[11] Vaca, C. E., Fang, J. L. & Schweda, E. K. Studies of the reaction of acetaldehyde with deoxynucleosides. *Chem. Biol. Interact.* **98**, 51–67 (1995). 7586051 10.1016/0009-2797(95)03632-v

[12] Wang, M. et al. Identification of DNA adducts of acetaldehyde. *Chem. Res Toxicol.* **13**, 1149–1157 (2000). 11087437 10.1021/tx000118t

[13] Brooks, P. J. & Theruvathu, J. A. DNA adducts from acetaldehyde: implications for alcohol-related carcinogenesis. *Alcohol* **35**, 187–193 (2005). 16054980 10.1016/j.alcohol.2005.03.009

[14] Garcia, C. C. et al. 13C2]-Acetaldehyde promotes unequivocal formation of 1,N2-propano-2’-deoxyguanosine in human cells. *J. Am. Chem. Soc.* **133**, 9140–9143 (2011). 21604744 10.1021/ja2004686

[15] Chang, J. et al. Genomic analysis of oesophageal squamous-cell carcinoma identifies alcohol drinking-related mutation signature and genomic alterations. *Nat. Commun.* **8**, 15290 (2017). 28548104 10.1038/ncomms15290 PMC5477513

[16] Li, X. C. et al. A mutational signature associated with alcohol consumption and prognostically significantly mutated driver genes in esophageal squamous cell carcinoma. *Ann. Oncol.* **29**, 938–944 (2018). 29351612 10.1093/annonc/mdy011 PMC5913594

[17] Wei, R. et al. Comprehensive analysis reveals distinct mutational signature and its mechanistic insights of alcohol consumption in human cancers. *Brief Bioinform.* **22**, bbaa066 (2021). 10.1093/bib/bbaa066 32480415

[18] Alexandrov, L. B. et al. The repertoire of mutational signatures in human cancer. *Nature* **578**, 94–101 (2020). 32025018 10.1038/s41586-020-1943-3 PMC7054213

[19] Plath, M. et al. Unraveling most abundant mutational signatures in head and neck cancer. *Int. J. Cancer* **148**, 115–127 (2021). 32930393 10.1002/ijc.33297

[20] Moody, S. et al. Mutational signatures in esophageal squamous cell carcinoma from eight countries with varying incidence. *Nat. Genet.* **53**, 1553–1563 (2021). 34663923 10.1038/s41588-021-00928-6

[21] Ristow, H. & Obe, G. Acetaldehyde induces cross-links in DNA and causes sister-chromatid exchanges in human cells. *Mutat. Res.* **58**, 115–119 (1978). 714076 10.1016/0165-1218(78)90103-9

[22] Clavijo-Cornejo, D. et al. Acetaldehyde targets superoxide dismutase 2 in liver cancer cells inducing transient enzyme impairment and a rapid transcriptional recovery. *Food Chem. Toxicol.* **69**, 102–108 (2014). 24746671 10.1016/j.fct.2014.04.002

[23] Harpaz, T. et al. The effect of ethanol on telomere dynamics and regulation in human cells. *Cells* **7**, 169 (2018). 10.3390/cells7100169 PMC6210749 30326633

[24] Kucab, J. E. et al. A Compendium of Mutational Signatures of Environmental Agents. *Cell* **177**, 821–836.e816 (2019). 30982602 10.1016/j.cell.2019.03.001 PMC6506336

[25] Tan, S. L. W. et al. A class of environmental and endogenous toxins induces BRCA2 haploinsufficiency and genome instability. *Cell* **169**, 1105–1118.e1115 (2017). 28575672 10.1016/j.cell.2017.05.010 PMC5457488

[26] Kotova, N. et al. Genotoxicity of alcohol is linked to DNA replication-associated damage and homologous recombination repair. *Carcinogenesis* **34**, 325–330 (2013). 23125219 10.1093/carcin/bgs340

[27] Helminen, A., Väkeväinen, S. & Salaspuro, M. ALDH2 genotype has no effect on salivary acetaldehyde without the presence of ethanol in the systemic circulation. *PLoS One* **8**, e74418 (2013). 24058561 10.1371/journal.pone.0074418 PMC3772811

[28] Linderborg, K., Salaspuro, M. & Väkeväinen, S. A single sip of a strong alcoholic beverage causes exposure to carcinogenic concentrations of acetaldehyde in the oral cavity. *Food Chem. Toxicol.* **49**, 2103–2106 (2011). 21641957 10.1016/j.fct.2011.05.024

[29] Salaspuro, M. Local Acetaldehyde: Its Key Role in Alcohol-Related Oropharyngeal Cancer. *Visc. Med* **36**, 167–173 (2020). 32775346 10.1159/000507234 PMC7383267

[30] Pastorino, R. et al. Effect of alcohol dehydrogenase-1B and -7 polymorphisms on blood ethanol and acetaldehyde concentrations in healthy subjects with a history of moderate alcohol consumption. *Drug Test. Anal.* **10**, 488–495 (2018). 28731573 10.1002/dta.2251

[31] Nass, F. et al. Influence of tiopronin on the metabolism of alcohol in healthy subjects. *Drug Res.* **67**, 204–210 (2017). 10.1055/s-0042-123826 28142160

[32] Liber, H. L. & Thilly, W. G. Mutation assay at the thymidine kinase locus in diploid human lymphoblasts. *Mutat. Res* **94**, 467–485 (1982). 6810168 10.1016/0027-5107(82)90308-6

[33] Szikriszt, B. et al. A comparative analysis of the mutagenicity of platinum-containing chemotherapeutic agents reveals direct and indirect mutagenic mechanisms. *Mutagenesis* **36**, 75–86 (2021). 33502495 10.1093/mutage/geab005 PMC8081379

[34] Martinek, R. et al. Comprehensive investigation of the mutagenic potential of six pesticides classified by IARC as probably carcinogenic to humans. *Chemosphere* **362**, 142700 (2024). 38936485 10.1016/j.chemosphere.2024.142700

[35] Farfán Labonne, B. E. et al. Acetaldehyde-induced mitochondrial dysfunction sensitizes hepatocytes to oxidative damage. *Cell Biol. Toxicol.* **25**, 599–609 (2009). 19137438 10.1007/s10565-008-9115-5

[36] Tamura, M., Ito, H., Matsui, H. & Hyodo, I. Acetaldehyde is an oxidative stressor for gastric epithelial cells. *J. Clin. Biochem Nutr.* **55**, 26–31 (2014). 25120276 10.3164/jcbn.14-12 PMC4078068

[37] Lózsa, R. et al. DNA mismatch repair protects the genome from oxygen-induced replicative mutagenesis. *Nucleic Acids Res.* **51**, 11040–11055 (2023). 37791890 10.1093/nar/gkad775 PMC10639081

[38] Németh, E. & Szüts, D. The mutagenic consequences of defective DNA repair. *DNA Repair* **139**, 103694 (2024). 38788323 10.1016/j.dnarep.2024.103694

[39] Yamazaki, K. et al. Homologous recombination contributes to the repair of acetaldehyde-induced DNA damage. *Cell Cycle* **23**, 369–384 (2024). 38571319 10.1080/15384101.2024.2335028 PMC11174073

[40] Kuykendall, J. R. & Bogdanffy, M. S. Reaction kinetics of DNA-histone crosslinking by vinyl acetate and acetaldehyde. *Carcinogenesis* **13**, 2095–2100 (1992). 1423881 10.1093/carcin/13.11.2095

[41] Oka, Y., Nakazawa, Y., Shimada, M. & Ogi, T. Endogenous aldehyde-induced DNA-protein crosslinks are resolved by transcription-coupled repair. *Nat. Cell Biol.* **26**, 784–796 (2024). 38600234 10.1038/s41556-024-01401-2 PMC11098742

[42] Stingele, J., Schwarz, M. S., Bloemeke, N., Wolf, P. G. & Jentsch, S. A DNA-dependent protease involved in DNA-protein crosslink repair. *Cell* **158**, 327–338 (2014). 24998930 10.1016/j.cell.2014.04.053

[43] Garaycoechea, J. I. et al. Alcohol and endogenous aldehydes damage chromosomes and mutate stem cells. *Nature* **553**, 171–177 (2018). 29323295 10.1038/nature25154 PMC6047743

[44] Bouwman, P. et al. A high-throughput functional complementation assay for classification of BRCA1 missense variants. *Cancer Discov.* **3**, 1142–1155 (2013). 23867111 10.1158/2159-8290.CD-13-0094

[45] Smith, J., Tho, L. M., Xu, N. & Gillespie, D. A. The ATM-Chk2 and ATR-Chk1 pathways in DNA damage signaling and cancer. *Adv. Cancer Res.* **108**, 73–112 (2010). 21034966 10.1016/B978-0-12-380888-2.00003-0

[46] Takahashi, S., Saito, S., Ohtani, N. & Sakai, T. Involvement of the Oct-1 regulatory element of the gadd45 promoter in the p53-independent response to ultraviolet irradiation. *Cancer Res.* **61**, 1187–1195 (2001). 11221850

[47] Dulić, V. et al. p53-dependent inhibition of cyclin-dependent kinase activities in human fibroblasts during radiation-induced G1 arrest. *Cell* **76**, 1013–1023 (1994). 8137420 10.1016/0092-8674(94)90379-4

[48] Scheer, M. A. et al. The involvement of acetaldehyde in ethanol-induced cell cycle impairment. *Biomolecules* **6**, 17 (2016). 10.3390/biom6020017 PMC4919912 27043646

[49] Tanaka, H. et al. A ribonucleotide reductase gene involved in a p53-dependent cell-cycle checkpoint for DNA damage. *Nature* **404**, 42–49 (2000). 10716435 10.1038/35003506

[50] Totoki, Y. et al. Multiancestry genomic and transcriptomic analysis of gastric cancer. *Nat. Genet.* **55**, 581–594 (2023). 36914835 10.1038/s41588-023-01333-x

[51] Suzuki, A. et al. Defined lifestyle and germline factors predispose Asian populations to gastric cancer. *Sci. Adv.* **6**, eaav9778 (2020). 32426482 10.1126/sciadv.aav9778 PMC7202881

[52] Saito-Adachi, M. et al. Oncogenic structural aberration landscape in gastric cancer genomes. *Nat. Commun.* **14**, 3688 (2023). 37349325 10.1038/s41467-023-39263-1 PMC10287692

[53] Freedman, N. D. et al. Alcohol intake and risk of oesophageal adenocarcinoma: a pooled analysis from the BEACON Consortium. *Gut* **60**, 1029–1037 (2011). 21406386 10.1136/gut.2010.233866 PMC3439838

[54] Ng, A. W. T. et al. Rearrangement processes and structural variations show evidence of selection in oesophageal adenocarcinomas. *Commun. Biol.* **5**, 335 (2022). 35396535 10.1038/s42003-022-03238-7 PMC8993906

[55] Moeller, B. C. et al. Biomarkers of exposure and effect in human lymphoblastoid TK6 cells following [13C2]-acetaldehyde exposure. *Toxicol. Sci.* **133**, 1–12 (2013). 23425604 10.1093/toxsci/kft029 PMC3627555

[56] Torrens, L. et al. The complexity of tobacco smoke-induced mutagenesis in head and neck cancer. *Nat. Genet* **57**, 884–896 (2025). 40164736 10.1038/s41588-025-02134-0 PMC11985354

[57] Smith, C. J. & Hansch, C. The relative toxicity of compounds in mainstream cigarette smoke condensate. *Food Chem. Toxicol.* **38**, 637–646 (2000). 10942325 10.1016/s0278-6915(00)00051-x

[58] Linhart, K., Bartsch, H. & Seitz, H. K. The role of reactive oxygen species (ROS) and cytochrome P-450 2E1 in the generation of carcinogenic etheno-DNA adducts. *Redox Biol.* **3**, 56–62 (2014). 25462066 10.1016/j.redox.2014.08.009 PMC4297928

[59] Chen, D. et al. BRCA1 deficiency specific base substitution mutagenesis is dependent on translesion synthesis and regulated by 53BP1. *Nat. Commun.* **13**, 226 (2022). 35017534 10.1038/s41467-021-27872-7 PMC8752635

[60] Murai, J. et al. Trapping of PARP1 and PARP2 by clinical PARP inhibitors. *Cancer Res.* **72**, 5588–5599 (2012). 23118055 10.1158/0008-5472.CAN-12-2753 PMC3528345

[61] Tacconi, E. M. et al. BRCA1 and BRCA2 tumor suppressors protect against endogenous acetaldehyde toxicity. *EMBO Mol. Med.* **9**, 1398–1414 (2017). 28729482 10.15252/emmm.201607446 PMC5623864

[62] Willis, N. A. et al. Mechanism of tandem duplication formation in BRCA1-mutant cells. *Nature* **551**, 590–595 (2017). 29168504 10.1038/nature24477 PMC5728692

[63] Kondratick, C. M., Washington, M. T. & Spies, M. Making choices: DNA replication fork recovery mechanisms. *Semin Cell Dev. Biol.* **113**, 27–37 (2021). 33967572 10.1016/j.semcdb.2020.10.001 PMC8098667

[64] Hoes, L. et al. Ethanol induces replication fork stalling and membrane stress in immortalized laryngeal cells. *iScience* **26**, 108564 (2023). 38213791 10.1016/j.isci.2023.108564 PMC10783606

[65] Langevin, F., Crossan, G. P., Rosado, I. V., Arends, M. J. & Patel, K. J. Fancd2 counteracts the toxic effects of naturally produced aldehydes in mice. *Nature* **475**, 53–58 (2011). 21734703 10.1038/nature10192

[66] Riva, L. et al. The mutational signature profile of known and suspected human carcinogens in mice. *Nat. Genet.* **52**, 1189–1197 (2020). 32989322 10.1038/s41588-020-0692-4 PMC7610456

[67] Hill, W. et al. Lung adenocarcinoma promotion by air pollutants. *Nature* **616**, 159–167 (2023). 37020004 10.1038/s41586-023-05874-3 PMC7614604

[68] Arakawa, H., Lodygin, D. & Buerstedde, J. M. Mutant loxP vectors for selectable marker recycle and conditional knock-outs. *BMC Biotechnol.* **1**, 7 (2001). 11591226 10.1186/1472-6750-1-7 PMC57747

[69] Guan, X., Rubin, E. & Anni, H. An optimized method for the measurement of acetaldehyde by high-performance liquid chromatography. *Alcohol Clin. Exp. Res.* **36**, 398–405 (2012). 21895715 10.1111/j.1530-0277.2011.01612.x PMC3235254

[70] Li, H. & Durbin, R. Fast and accurate short read alignment with Burrows-Wheeler transform. *Bioinformatics* **25**, 1754–1760 (2009). 19451168 10.1093/bioinformatics/btp324 PMC2705234

[71] McKenna, A. et al. The genome analysis toolkit: a mapreduce framework for analyzing next-generation DNA sequencing data. *Genome Res.* **20**, 1297–1303 (2010). 20644199 10.1101/gr.107524.110 PMC2928508

[72] Pipek, O. et al. Fast and accurate mutation detection in whole genome sequences of multiple isogenic samples with IsoMut. *BMC Bioinforma.* **18**, 73 (2017). 10.1186/s12859-017-1492-4 PMC5282906 28143617

[73] Zamborszky, J. et al. Loss of BRCA1 or BRCA2 markedly increases the rate of base substitution mutagenesis and has distinct effects on genomic deletions. *Oncogene* **36**, 746–755 (2017). 27452521 10.1038/onc.2016.243 PMC5096687

[74] Szikriszt, B. et al. A comprehensive survey of the mutagenic impact of common cancer cytotoxics. *Genome Biol.* **17**, 99 (2016). 27161042 10.1186/s13059-016-0963-7 PMC4862131

[75] Cameron, D. L. et al. GRIDSS2: comprehensive characterisation of somatic structural variation using single breakend variants and structural variant phasing. *Genome Biol.* **22**, 202 (2021). 34253237 10.1186/s13059-021-02423-x PMC8274009

[76] Manders, F. et al. MutationalPatterns: the one stop shop for the analysis of mutational processes. *BMC Genomics* **23**, 134 (2022). 35168570 10.1186/s12864-022-08357-3 PMC8845394

[77] Bray, N. L., Pimentel, H., Melsted, P. & Pachter, L. Near-optimal probabilistic RNA-seq quantification. *Nat. Biotechnol.* **34**, 525–527 (2016). 27043002 10.1038/nbt.3519

[78] Soneson, C., Love, M. I. & Robinson, M. D. Differential analyses for RNA-seq: transcript-level estimates improve gene-level inferences. *F1000Res* **4**, 1521 (2015). 26925227 10.12688/f1000research.7563.1 PMC4712774

[79] Love, M. I., Huber, W. & Anders, S. Moderated estimation of fold change and dispersion for RNA-seq data with DESeq2. *Genome Biol.* **15**, 550 (2014). 25516281 10.1186/s13059-014-0550-8 PMC4302049

[80] Zhu, A., Ibrahim, J. G. & Love, M. I. Heavy-tailed prior distributions for sequence count data: removing the noise and preserving large differences. *Bioinformatics* **35**, 2084–2092 (2019). 30395178 10.1093/bioinformatics/bty895 PMC6581436

[81] Kim, D., Paggi, J. M., Park, C., Bennett, C. & Salzberg, S. L. Graph-based genome alignment and genotyping with HISAT2 and HISAT-genotype. *Nat. Biotechnol.* **37**, 907–915 (2019). 31375807 10.1038/s41587-019-0201-4 PMC7605509

[82] Xu, S. et al. Using clusterProfiler to characterize multiomics data. *Nat. Protoc.* **19**, 3292–3320 (2024). 39019974 10.1038/s41596-024-01020-z

[83] Zhang, J. et al. The International Cancer Genome Consortium Data Portal. *Nat. Biotechnol.* **37**, 367–369 (2019). 30877282 10.1038/s41587-019-0055-9

