# Supplementary material for: Long-term exposure to the ethanol-derived metabolite acetaldehyde elevates structural genomic alterations but not base substitutions
Source: Commun Biol. 2026 Jan 17;9:243. doi: 10.1038/s42003-026-09521-1 (PMC12905380; doi:10.1038/s42003-026-09521-1)
Supplement: Supplementary file 1 — Supplemental Information [file 42003_2026_9521_MOESM1_ESM.pdf]

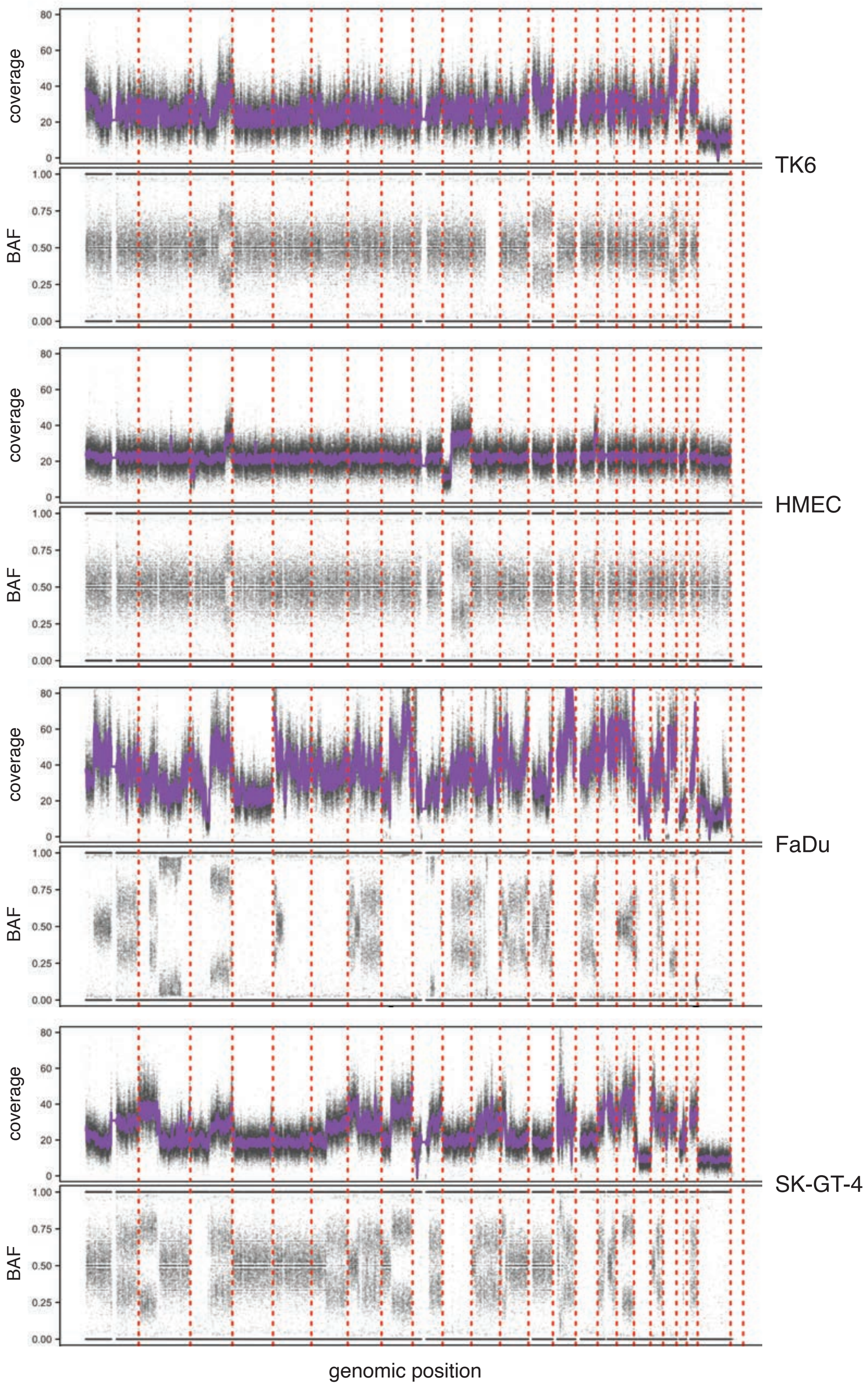

Supplementary Figure S1. Chromosome copy number variations in the ancestral clones of each cell line.

Sequence coverage of genomic positions with a sliding average in purple (top panel) and allele frequency of selected typically heterozygous human SNPs (B allele frequency, BAF, bottom panel) are shown for each ancestral clone. Chromosome boundaries are indicated, human chromosomes are shown in numerical order.

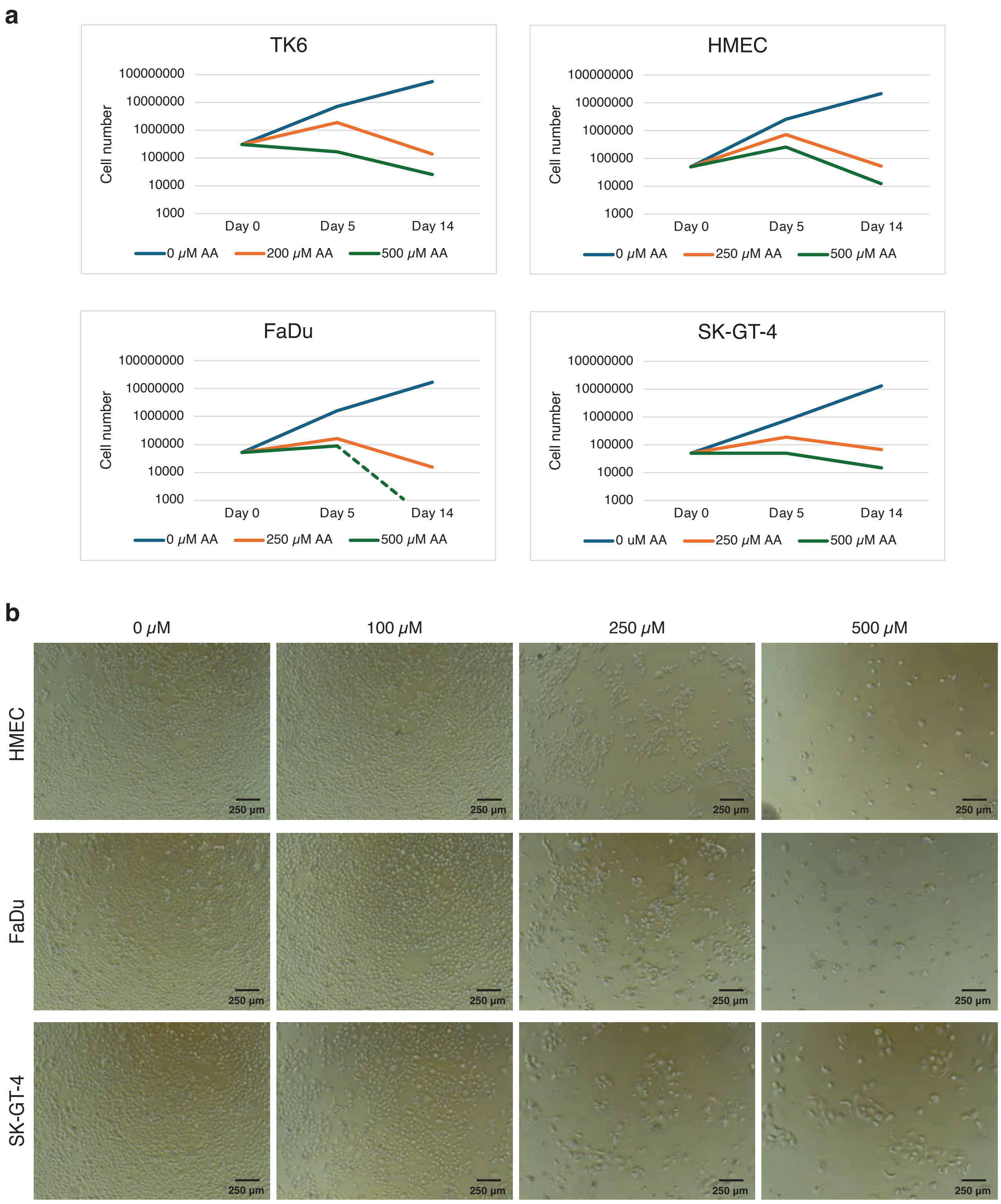

Supplementary Figure S2. Selection of long-term treatment concentration
(a) Cell numbers in cultures of the cell lines shown above the panel, when treated with the indicated concentrations of acetaldehyde (AA). (b) Images of adherent cell types treated with the indicated concentrations of acetaldehyde for 5 days (HMEC) or 7 days (FaDu and SK-GT-4). 100 μM concentration was chosen for long-term treatments as the cell lines did not survive higher tested concentrations.

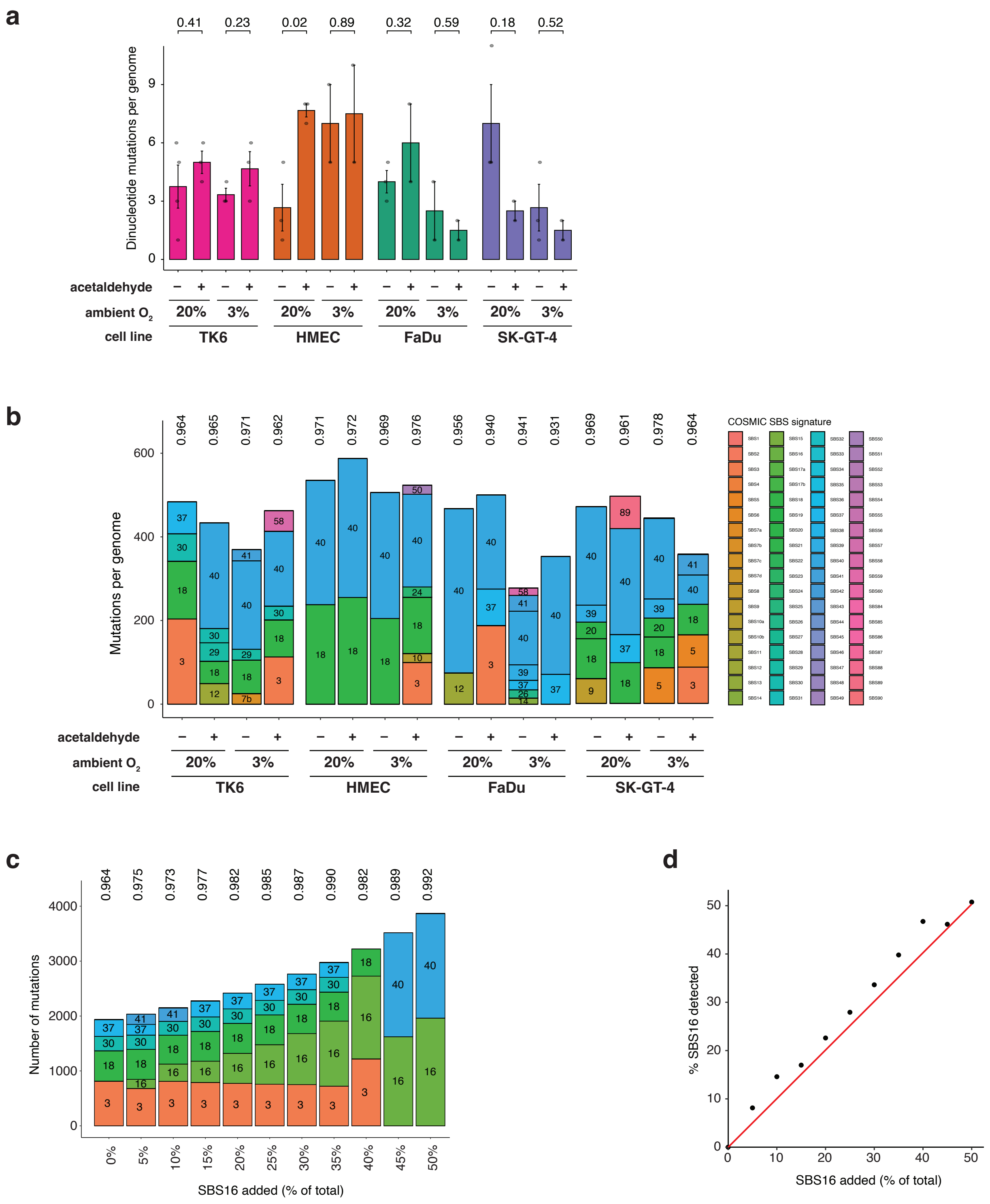

Supplementary Figure S3. Dinucleotide mutations and SBS signature deconstruction

(a) Dinucleotide base substitution mutations in the whole genome sequenced samples. Values for individual samples are indicated with black markers, error bars show SEM. The significance values of pairwise comparisons is indicated (unpaired two-sided t test, no correction is applied for multiple comparisons). (b) Deconstruction of the experimental SBS mutation spectra determined from the average of the replicate samples (Fig. 2d) to the indicated COSMIC SBS mutation signatures. (c) Sensitivity of detection of SBS16. Randomly generated mutations with an SBS16 spectrum were added to the total TK6 mock treated dataset to contribute the indicated percentage of the final mutation pool, which was then deconstructed to COSMIC signatures. As little as 5% contribution can be detected. Deconstructions show an instability between SBS3 and SBS40 in (c, d). (d) Percentage of SBS16 mutations added in (c) and found by deconstructing the same mutation spectra.

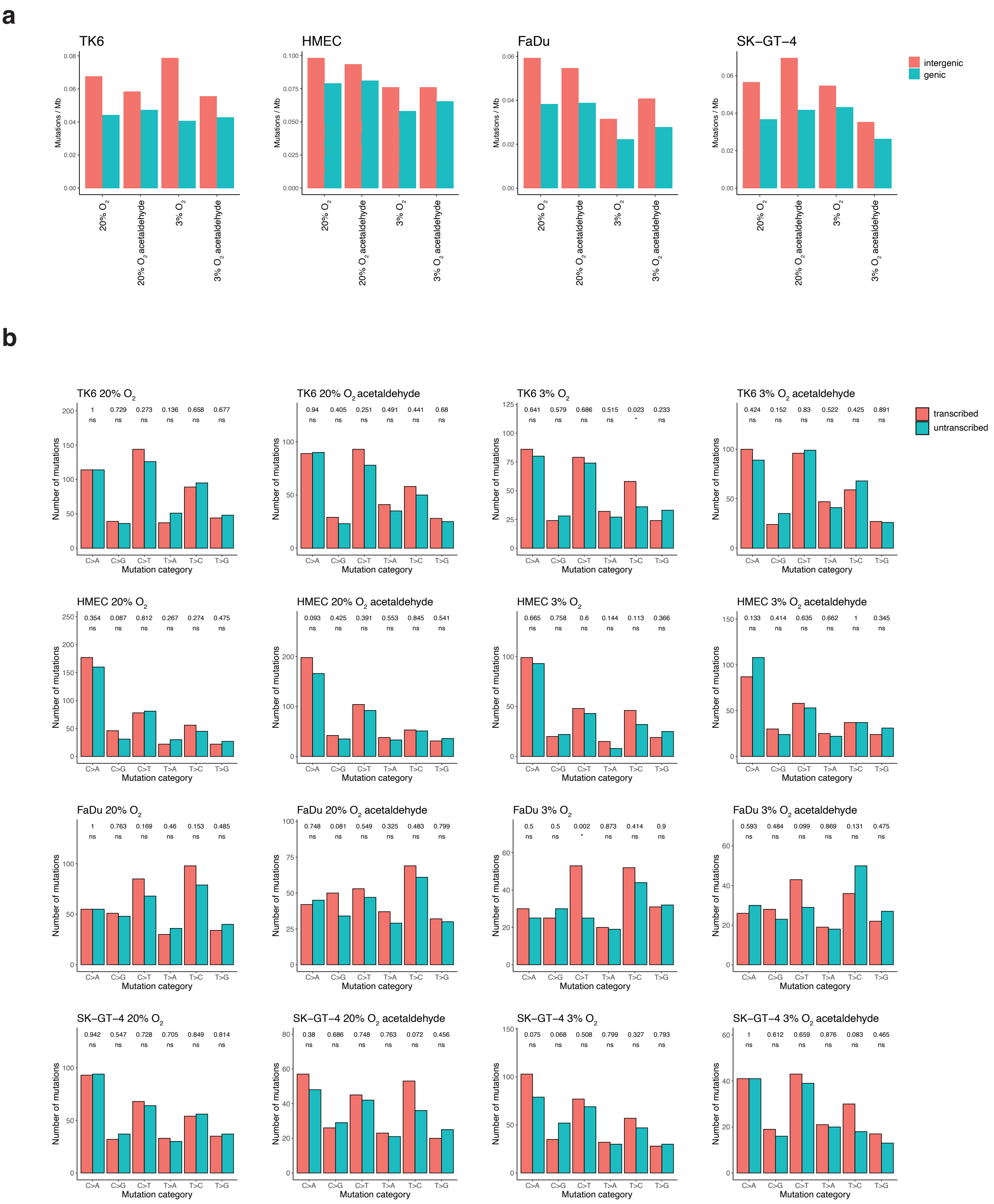

Supplementary Figure S4. Genomic distribution of SBS mutations

(a) SBS mutation density in genic and intergenic regions, shown by cell line and treatment. (b) Transcriptional strand bias of SBS mutations. Panels show the total number of mutations on the transcribed and untranscribed strand of genic regions, separated by mutation category, for each set of parallel samples. Significant differences between the strands is shown above the bars (* $p<0.05$, χ^2 test, no correction for multiple comparisons, ns not significant).

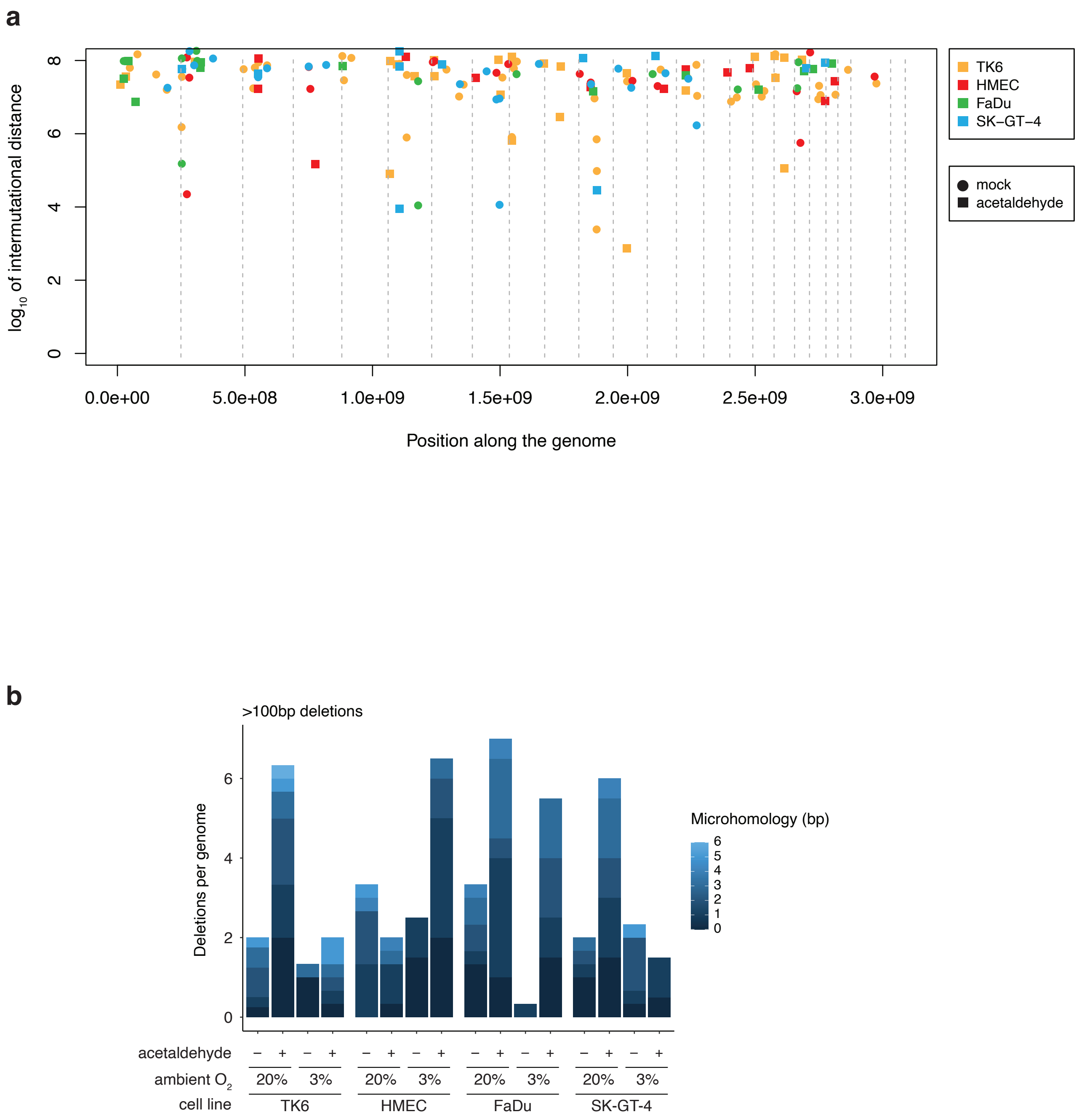

Supplementary Figure S5. Properties of structural variations

(a) Rainfall plot of the genomic positions of all structural variation events, presented along the human genome (*x* axis, dashed lines indicate chromosome boundaries) at the midpoint position between the two breakpoints. The *y* axis shows the distance of each event from the previous one across the whole dataset. Some mild clustering is observable, independent of cell line or treatment. (b) Analysis of microhomology between the breakpoints of large deletions over 100 bp.

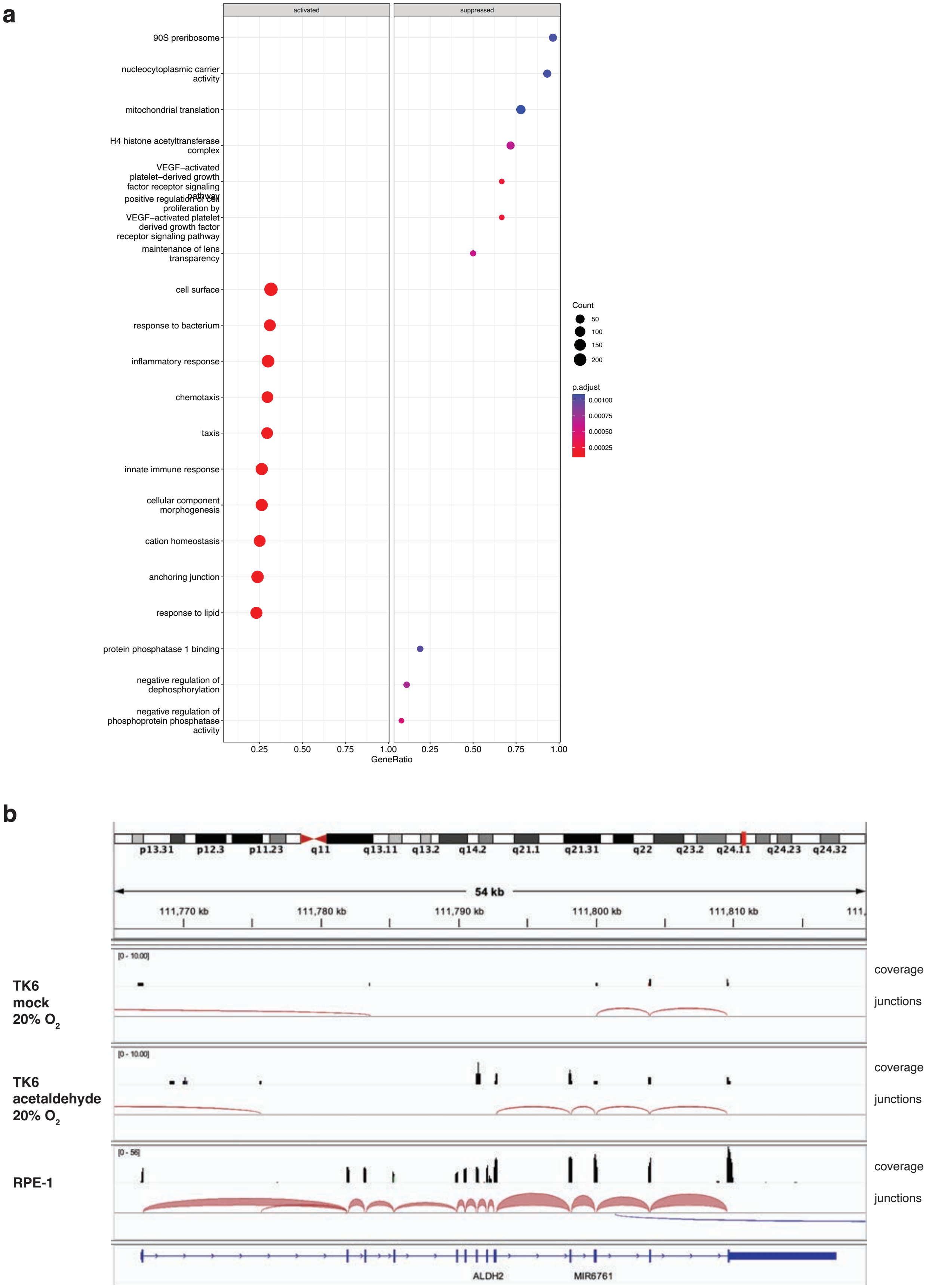

Supplementary Figure S6. Gene expression changes upon 100 µM acetaldehyde treatment of TK6 cells for 7 days.

(a) Gene ontology analysis of gene expression changes at the pathway level. (b) Screenshot from the Integrative Genomics Viewer (IGV) software showing a lack of *ALDH2* expression in TK6 cells with or without acetaldehyde treatment. A sample of untreated RPE-1 cells is shown for comparison.

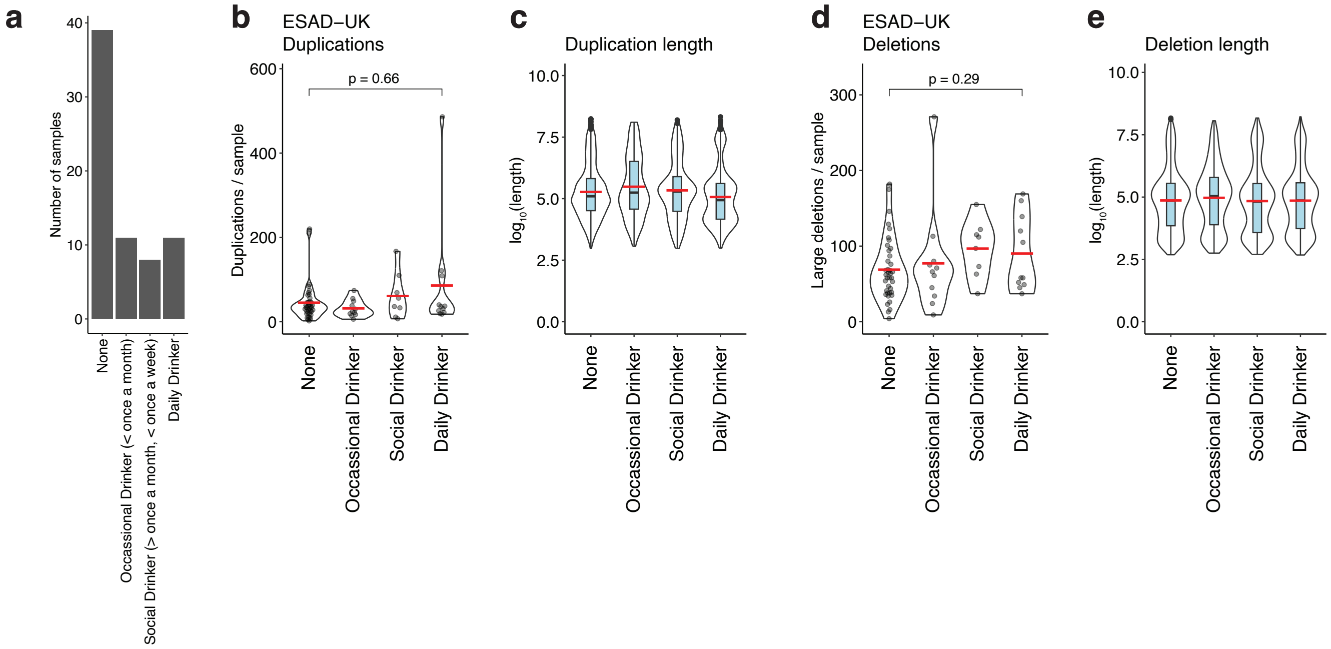

Supplementary Figure S7. Structural variations in esophageal adenocarcinomas

(a) The number of samples in the ESAD-UK dataset categorised by the history of alcohol consumption. (b) Duplications per sample in each category. (c) The length distribution of duplications; box plots show median and interquartile range. (d) Large deletions per sample in each category. (e) The length distribution of deletions; box plots show median and interquartile range. Mean values are shown in red in panels (b-e). The significance value of the difference between the number of events in non-drinkers and daily drinkers is shown in (b) and (d), Wilcoxon rank-sum test.

a

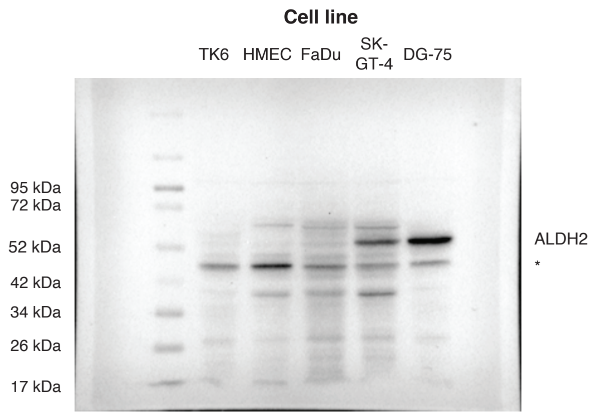

b

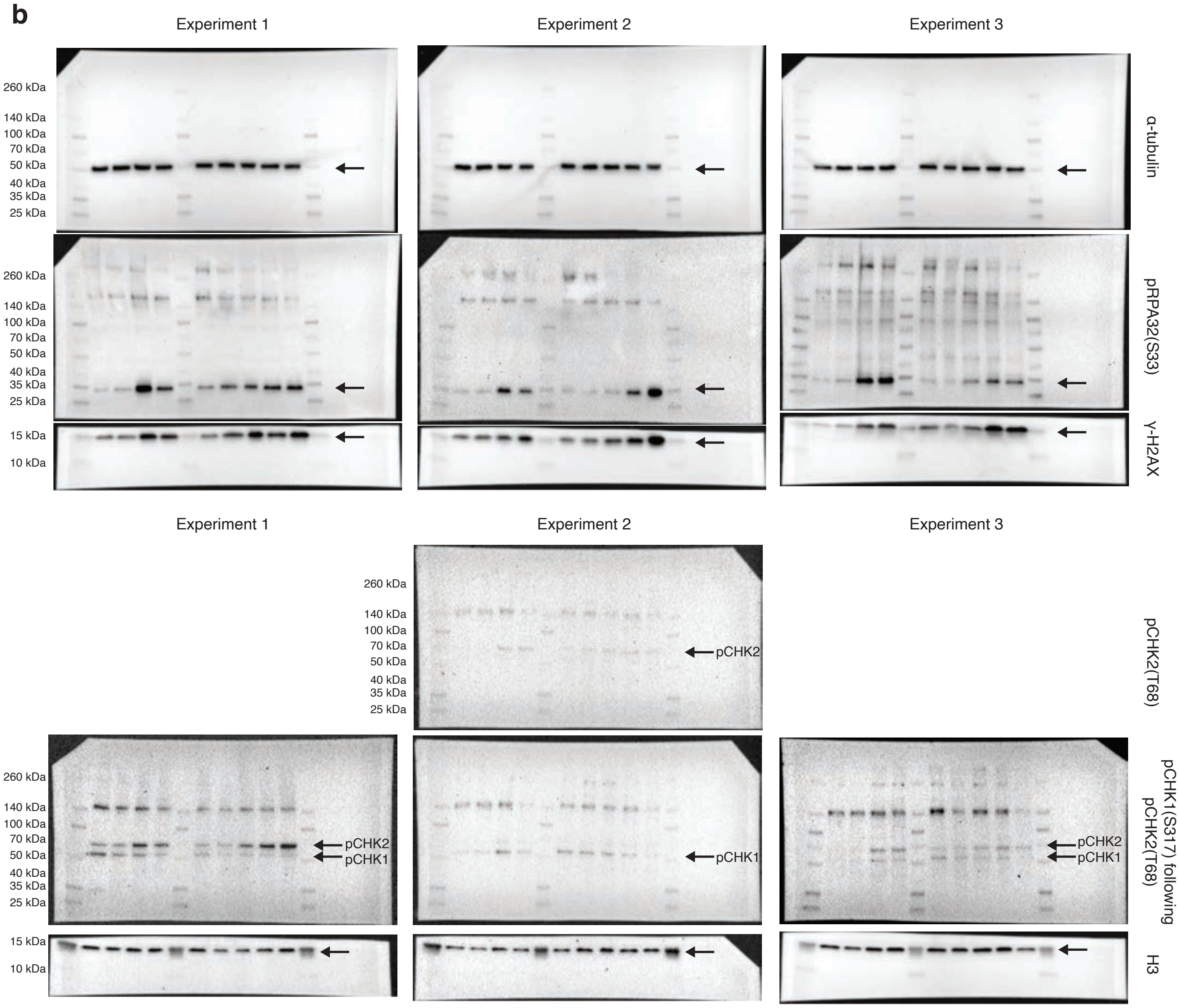

Supplementary Figure S8. Uncropped Western blot images.

(a) Uncropped blot for Fig. 1d. (b) Uncropped blots for Fig. 5a, and further blots used for quantification in Fig. 5b. In Experiment 2, pCHK2 quantification was performed on a separate, earlier labelling (top panel). The sample order is as presented on Fig. 5a. Arrows indicate the quantified bands.
