# Supplementary material for: Long-term exposure to the ethanol-derived metabolite acetaldehyde elevates structural genomic alterations but not base substitutions
Source: Commun Biol. 2026 Jan 17;9:243. doi: 10.1038/s42003-026-09521-1 (PMC12905380; doi:10.1038/s42003-026-09521-1)
Supplement: Supplementary file 2 — Description of Additional Supplementary Files [file 42003_2026_9521_MOESM2_ESM.docx]

**Description of Additional Supplementary File**

 File name: Supplementary Data 1
Description: Primers used for this study

File name: Supplementary Data 2
Description: Lists of identified mutations in whole genome sequencing data

File name: Supplementary Data 3
Description: Source data for all figures
