# Supplementary material for: Long-term exposure to the ethanol-derived metabolite acetaldehyde elevates structural genomic alterations but not base substitutions
Source: Commun Biol. 2026 Jan 17;9:243. doi: 10.1038/s42003-026-09521-1 (PMC12905380; doi:10.1038/s42003-026-09521-1)
Supplement: Supplementary file 6 — Reporting summary [file 42003_2026_9521_MOESM6_ESM.pdf]

nature portfolio

Corresponding author(s): David Szuts

Last updated by author(s): 18 December 2025

Reporting Summary

Nature Portfolio wishes to improve the reproducibility of the work that we publish. This form provides structure for consistency and transparency in reporting. For further information on Nature Portfolio policies, see our Editorial Policies and the Editorial Policy Checklist.

Statistics

For all statistical analyses, confirm that the following items are present in the figure legend, table legend, main text, or Methods section.

n/a	Confirmed	
☐	☒	The exact sample size (*n*) for each experimental group/condition, given as a discrete number and unit of measurement
☐	☒	A statement on whether measurements were taken from distinct samples or whether the same sample was measured repeatedly
☐	☒	The statistical test(s) used AND whether they are one- or two-sided<br>*Only common tests should be described solely by name; describe more complex techniques in the Methods section.*
☒	☐	A description of all covariates tested
☐	☒	A description of any assumptions or corrections, such as tests of normality and adjustment for multiple comparisons
☐	☒	A full description of the statistical parameters including central tendency (e.g. means) or other basic estimates (e.g. regression coefficient) AND variation (e.g. standard deviation) or associated estimates of uncertainty (e.g. confidence intervals)
☐	☒	For null hypothesis testing, the test statistic (e.g. *F*, *t*, *r*) with confidence intervals, effect sizes, degrees of freedom and *P* value noted<br>*Give P values as exact values whenever suitable.*
☒	☐	For Bayesian analysis, information on the choice of priors and Markov chain Monte Carlo settings
☒	☐	For hierarchical and complex designs, identification of the appropriate level for tests and full reporting of outcomes
☒	☐	Estimates of effect sizes (e.g. Cohen's *d*, Pearson's *r*), indicating how they were calculated

Our web collection on statistics for biologists contains articles on many of the points above.

Software and code

Policy information about availability of computer code

Data collection	No software was used for data collection.
Data analysis	The use of genomics software tools is specified and referenced in the Methods: "Sequencing reads were aligned to the reference genome GRCh38/hg38 using the Burrows-Wheeler alignment algorithm ref70, followed by post-processing with the IndelRealigner tool of the Genome analysis Toolkit (GATK, version 3.8) ref71. Single base substitution (SBS) and short indel (<50bp) mutations were identified using the IsoMut method developed for multiple isogenic samples ref72-74 "SVs were detected using GRIDSS v2.8.3 ref75" (...)<br>Abundances of transcripts were calculated using kallisto 0.48.0 ref77 from fastq files, and were imported and summarised with tximport ref78. Genes were annotated based on Ensembl genes build GRCh38.111. Differentially expressed genes were determined with DESeq2 ref79. Approximate Posterior Estimation for generalised linear model (R package Apeglm ref80) was used for log fold change shrinkage to reduce noise of low expression genes.

For manuscripts utilizing custom algorithms or software that are central to the research but not yet described in published literature, software must be made available to editors and reviewers. We strongly encourage code deposition in a community repository (e.g. GitHub). See the Nature Portfolio guidelines for submitting code & software for further information.

Data

Policy information about availability of data

All manuscripts must include a data availability statement. This statement should provide the following information, where applicable:

- Accession codes, unique identifiers, or web links for publicly available datasets
- A description of any restrictions on data availability
- For clinical datasets or third party data, please ensure that the statement adheres to our policy

Source data for all figures is provided in Supplementary Data 3. Whole genome sequence and transcriptome data obtained for this study is available from the European Nucleotide Archive under study accession number PRJEB81715.

Research involving human participants, their data, or biological material

Policy information about studies with human participants or human data. See also policy information about sex, gender (identity/presentation), and sexual orientation and race, ethnicity and racism.

Reporting on sex and gender	Published human cancer genomics datasets were analysed for this study. Sex and gender were not part of the dataset.
Reporting on race, ethnicity, or other socially relevant groupings	Published human cancer genomics datasets were analysed for this study. Ethnicity and social attributes were not part of the dataset.
Population characteristics	Covariates were not analysed. Cancer genomics data were obtained from two different published sources: ICGC (International Cancer Genome Consortium, Fig. S2) and a separate dataset published in ref52. The two datasets did not provide comparable covariate information.
Recruitment	No participant recruitment was done in this study.
Ethics oversight	Only previously published, public data were analysed.

Note that full information on the approval of the study protocol must also be provided in the manuscript.

Field-specific reporting

Please select the one below that is the best fit for your research. If you are not sure, read the appropriate sections before making your selection.

☒ Life sciences ☐ Behavioural & social sciences ☐ Ecological, evolutionary & environmental sciences

For a reference copy of the document with all sections, see nature.com/documents/nr-reporting-summary-flat.pdf

Life sciences study design

All studies must disclose on these points even when the disclosure is negative.

Sample size	Sample sizes for genomic analyses were almost universally n=3 independent biological cell clones, chosen mainly for cost considerations. n=3 was sufficient to show significant differences upon diverse biological conditions in mutation numbers in our previous, relevant publications (upon environmental mutagen treatment: Szikriszt et al., 2016, Martinek et al, 2024; these publications are cited in the manuscript) therefore it was appropriate to use the same sample sizes for the presented related experiments.<br>For experiments presented in figure 4A, the sample number was n=4. The standard deviations and the expected effects were not known in advance, therefore this sample number was chosen as a uniform standard in advance and only those differences were reported as significant which had p<0.05 with two-sided unpaired t-tests. The experiments presented in figure 5A and 5B were repeated three times, a representative image is shown.
Data exclusions	Whole genome sequenced samples were excluded when they were not independent from each other, i.e. when mutation detection found mostly the same mutations in a pair of samples, one of the pair was excluded.
Replication	Whole genome sequencing based experiments were performed on a single occasion with three parallel biological replicates (individual cell clones from different parallel cultures were sequenced). Cytotoxicity replica measurement were performed over a long time period, and showed good reproducibility with no observable trend in variation.
Randomization	Our study did not involve allocation of samples to different experimental groups. Randomisation is therefore not relevant. When treated and not treated populations or cell clones were compared, these were based on the bulk separation of an original starting population, not on the allocation of individual samples to experimental groups.
Blinding	All bioinformatics analyses (mutation detection) are exactly reproducible, therefore blinding was not necessary. Nevertheless, the software methods do not require an input about the identity of the samples (e.g. which sequenced genomes were treated), therefore in essence the analyses were blinded. Cytotoxicity measurements were performed by an automated plate reader.<br>All microscope preparations were blinded before counting of sister chromatid exchanges.

Reporting for specific materials, systems and methods

We require information from authors about some types of materials, experimental systems and methods used in many studies. Here, indicate whether each material, system or method listed is relevant to your study. If you are not sure if a list item applies to your research, read the appropriate section before selecting a response.

Materials & experimental systems

n/a	Involved in the study
☐	☒ Antibodies
☐	☒ Eukaryotic cell lines
☒	☐ Palaeontology and archaeology
☒	☐ Animals and other organisms
☒	☐ Clinical data
☒	☐ Dual use research of concern
☒	☐ Plants

Methods

n/a	Involved in the study
☒	☐ ChIP-seq
☒	☐ Flow cytometry
☒	☐ MRI-based neuroimaging

Antibodies

Antibodies used	Anti-ALDH2 antibody (Proteintech Europe, Manchester, UK, #15310-1-AP, lot 0016948); phospho-RPA32 (Ser33) antibody (Santa Cruz Biotechnology, Texas, USA, #sc-28709); phospho-histone H2A.X (Ser139) antibody (Sigma-Aldrich, Missouri, USA, #ZMS05636); phospho-Chk1 (Ser317) antibody (Cell Signaling Technology, Leiden, The Netherlands, #2344S); phospho-Chk2 (Thr68) antibody (Cell Signaling Technology, Leiden, The Netherlands, #2661), H3-histone antibody (Santa Cruz Biotechnology, Dallas, USA, #sc-10809) and α-tubulin antibody (Merck, Darmstadt, Germany, #T6199)
Validation	Anti-ALDH2 antibody: The manufacturer recommends it for use on human cell lysates. A band of the expected 52 kDa size correlated with the expression level of ALDH2 based on transcriptome data (see manuscript text). A band of this size disappeared upon ALDH2 silencing in Yang et al 2021, Cancer Discov, doi:10.1158/2159-8290.CD-20-1542 when detected with the same antibody. The phospho-histone H2A.X (Ser139) antibody was raised against a conjugated linear peptide corresponding to 9 amino acids surrounding phosphoserine 139, and only recognizes one protein (shows one band) at the expected size of histone H2AX. The phospho-RPA32 (Ser133) antibody was raised against a phosphorylated synthetic peptide, which represented a portion of human replication protein A2, 32 kDa surrounding phosphorylated serine that corresponded to position 33, and only recognizes one protein (shows one band) at the expected size of pRPA32 (33kDa). The phospho-CHK1 (Ser317) antibody only recognised one band in the expected size range of 50 kDa. The phospho-CHK2 (Thr68) antibody only recognised one band in the expected size range of 60 kDa, and its intensity correlated with DNA damage shown by independent experiments in this study. H3-histone antibody (Santa Cruz Biotechnology, Dallas, USA, #sc-10809) was used as loading control and only recognised one band in the expected size range of 15 kDa. α-tubulin antibody (Merck, Darmstadt, Germany, #T6199) was used as loading control and only recognised one band in the expected size range of 50-55 kDa.

Eukaryotic cell lines

Policy information about cell lines and Sex and Gender in Research

Cell line source(s)	Four human cell lines were used in this study, which were purchased from the sources shown below.<br>TK6: JCRB, Ibaraki, Japan, #JCRB1435 - male<br>FaDu: LGC Standards, Teddington, Middlesex, UK, #ATCC-HTB-43 - male<br>SK-GT-4: Merck, Darmstadt, Germany, #11012007 - male<br>hTERT-HMEC: Clonetech, Mountain View, CA, US - female<br><br>The chicken DT40 cell line was also used (female). DT40 cell lines were derived from sequenced, validated clones in the group's previous publications (doi: 0.1038/s41467-021-27872-7 and 10.1186/s13059-019-1867-0) or made for this study as described in the methods section.
Authentication	All cell lines were authenticated using the whole genome sequencing data.
Mycoplasma contamination	The cell lines were tested and found negative for mycoplasma contamination.
Commonly misidentified lines<br>(See ICLAC register)	No such lines were used.

Plants

Seed stocks	NA.
Novel plant genotypes	*Describe the methods by which all novel plant genotypes were produced. This includes those generated by transgenic approaches, gene editing, chemical/radiation-based mutagenesis and hybridization. For transgenic lines, describe the transformation method, the number of independent lines analyzed and the generation upon which experiments were performed. For gene-edited lines, describe the editor used, the endogenous sequence targeted for editing, the targeting guide RNA sequence (if applicable) and how the editor was applied.*
Authentication	*Describe any authentication procedures for each seed stock used or novel genotype generated. Describe any experiments used to assess the effect of a mutation and, where applicable, how potential secondary effects (e.g. second site T-DNA insertions, mosiacism, off-target gene editing) were examined.*
